# Novel materials in magnetic resonance imaging: high permittivity ceramics, metamaterials, metasurfaces and artificial dielectrics

**DOI:** 10.1007/s10334-022-01007-5

**Published:** 2022-04-26

**Authors:** Andrew Webb, Alena Shchelokova, Alexey Slobozhanyuk, Irena Zivkovic, Rita Schmidt

**Affiliations:** 1 Department of Radiology, C.J. Gorter MRI Centre, Leiden University Medical Center, Leiden, The Netherlands; 2 School of Physics and Engineering, ITMO University, Saint Petersburg, Russia; 3 Department of Electrical Engineering, TU Eindhoven, Eindhoven, The Netherlands; 4 Department of Brain Sciences, Weizmann Institute of Science, Rehovot, Israel

**Keywords:** High permittivity materials, Dielectrics, Transmit efficiency, Metamaterials, Metasurfaces, Artificial dielectrics

## Abstract

This article reviews recent developments in designing and testing new types of materials which can be: (i) placed around the body for in vivo imaging, (ii) be integrated into a conventional RF coil, or (iii) form the resonator itself. These materials can improve the quality of MRI scans for both in vivo and magnetic resonance microscopy applications. The methodological section covers the basic operation and design of two different types of materials, namely high permittivity materials constructed from ceramics and artificial dielectrics/metasurfaces formed by coupled conductive subunits, either in air or surrounded by dielectric material. Applications of high permittivity materials and metasurfaces placed next to the body to neuroimaging and extremity imaging at 7 T, body and neuroimaging at 3 T, and extremity imaging at 1.5 T are shown. Results using ceramic resonators for both high field in vivo imaging and magnetic resonance microscopy are also shown. The development of new materials to improve MR image quality remains an active area of research, but has not yet found significant use in clinical applications. This is mainly due to practical issues such as specific absorption rate modelling, accurate and reproducible placement, and acceptable size/weight of such materials. The most successful area has been simple “dielectric pads” for neuroimaging at 7 T which were initially developed somewhat as a stop-gap while parallel transmit technology was being developed, but have continued to be used at many sites. Some of these issues can potentially be overcome using much lighter metasurfaces and artificial dielectrics, which are just beginning to be assessed.

## Introduction

When a whole-body 7 T Philips MRI system was installed in the Department of Radiology in Leiden in 2007, it quickly became clear that to get clinicians on board, the image quality using almost every sequence would have to be improved substantially. Signal drop outs in the temporal lobes in T_2_-weighted scans were particularly evident, and the gradual loss of signal in the head/foot direction in the posterior part of the cerebellum and flat contrast in the same area were off-putting. This was not a surprise: much seminal work had predicted that there would be both advantageous and disadvantageous aspects of imaging at frequencies where the wavelength and body dimensions become comparable [1–3]. Although as physicists we tend to accept what we understand as shortcomings, the wide variation in image contrast across an image was very unfamiliar to neuroradiologists, and so reduced their level of confidence for diagnostic use. The high field group in Minnesota [4] had already shown that a parallel transmit approach could be used to mitigate the transmit field (B_1_^+^) inhomogeneity, but this hardware was not widely available at that time. Having worked with high permittivity materials (HPMs) in resonator designs for high field magnetic resonance microscopy, the fact that these materials redistribute the magnetic and electric field distributions seems like an interesting way to approach this problem, with a subtle difference being that specific modes of the material would not be used, since this would concentrate the field inside the material, but rather use the displacement currents induced by the primary transmit coil to produce secondary magnetic fields to add constructively with the primary field. Earlier work by Alsop [5] and in particular Yang [6] had showed that large bags of water placed around the head could produce such a redistribution of electromagnetic (EM) fields. However, for realistic applications these were much too bulky: the material needed to fit inside a (then) 16-channel and (now) 32-channel receive array to enable full parallel imaging performance. The solution was to produce thin pads which were made from high permittivity metal titanates, with the gaps between particles filled with a high permittivity liquid (water or deuterated water) [7]; this formulation has the advantage of also being relatively malleable for placing around the subject’s head. This approach was developed very much with a “stop-gap” mentality; it gave substantial image quality improvement, was much appreciated by radiologists, and was simple enough and importantly vendor-independent such that it could be used by many of the fledgling high field sites around the world. However, it was fully anticipated that parallel-transmit approaches would slowly be introduced and would supplant this more simple approach, or perhaps a combination of the two would turn out to be the optimal solution.

While waiting for this to happen, we and a few other groups started to apply this approach to clinical field strengths i.e. 3 T or even 1.5 T, especially for older MR systems in less economically well-off parts of the world. In this regards, it was clear that new materials would have to be designed. Similar ideas were being formulated and published by the Materials Science group at the Pennsylvania State University in the USA, working with the Collins group in New York University. These approaches concentrated on high density ceramics with very high permittivities, using specialized materials. We also started to look at alternative structures such as metasurfaces, metamaterials and artificial dielectrics which might provide equivalent permittivities, but would be much lighter. Much of this work was performed in collaboration with ITMO in St. Petersburg in Russia. Having started to use such materials for in vivo use, it also became clear that these specialized materials could be used to make very efficient resonators for very high field magnetic resonance microscopy, thus in a way completing a circle of development ideas.

## Spatially inhomogeneous transmit and receive fields

**Fig. 1:**
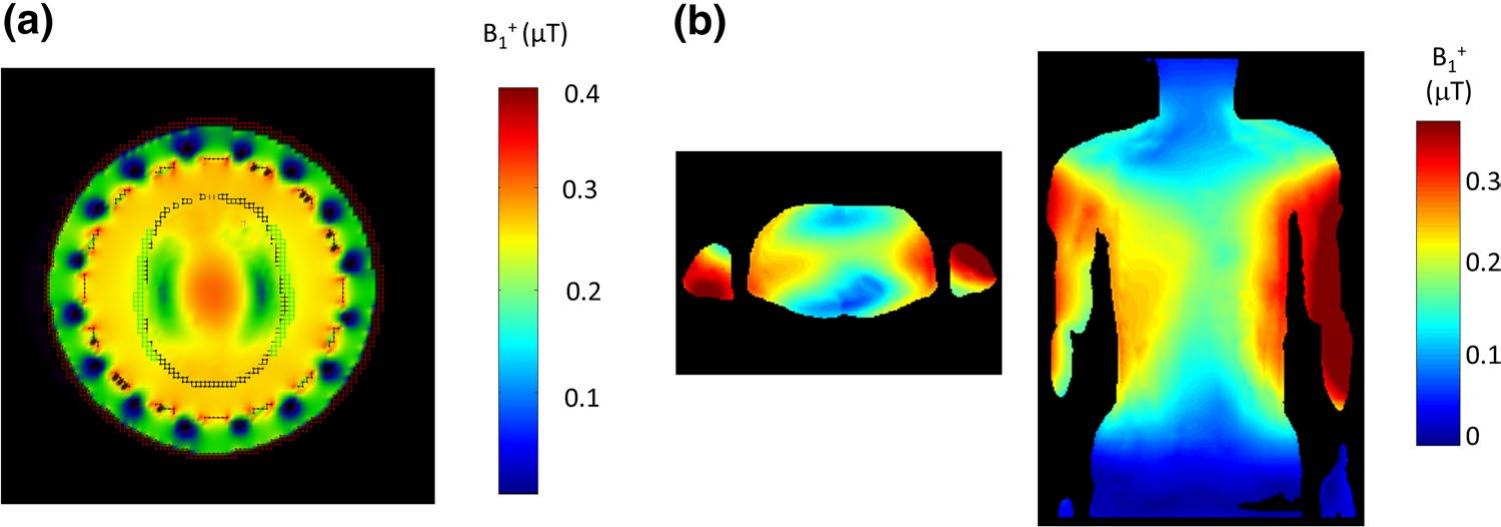
**a** Simulated B_1_^+^ field from a circularly polarized birdcage coil loaded with the human model Duke at 7 T: the image is an axial slice through the centre of the brain. B_1_^+^ values are normalized with respect to 1 Watt dissipated power. Figure reproduced from [8]. **b** Simulated B_1_^+^ fields through the centre of the liver in Duke at 3 T in axial and coronal views. B_1_^+^ values are normalized with respect to 1 Watt dissipated power. Figure reproduced from [9]

Why might one need additional materials placed around the head or body to improve image quality? What is the problem that needs to be addressed? Consider the transmit (B_1_^+^) field distribution at 7 T in the head produced by a circularly-polarized birdcage head coil, shown in Fig. 1a. There is very low transmit efficiency in the area around the temporal lobes, with a very high efficiency at the centre of the brain. Figure 1b shows the situation for body imaging at 3 T, which shows a somewhat similar B_1_^+^ distribution rotated by 90° due to the rotated ellipticity of the object. In this case the “central brightening” is mitigated by a greater absorption of the transmit field due to the much larger amount of conductive tissue through which it must pass.

**Fig. 2:**
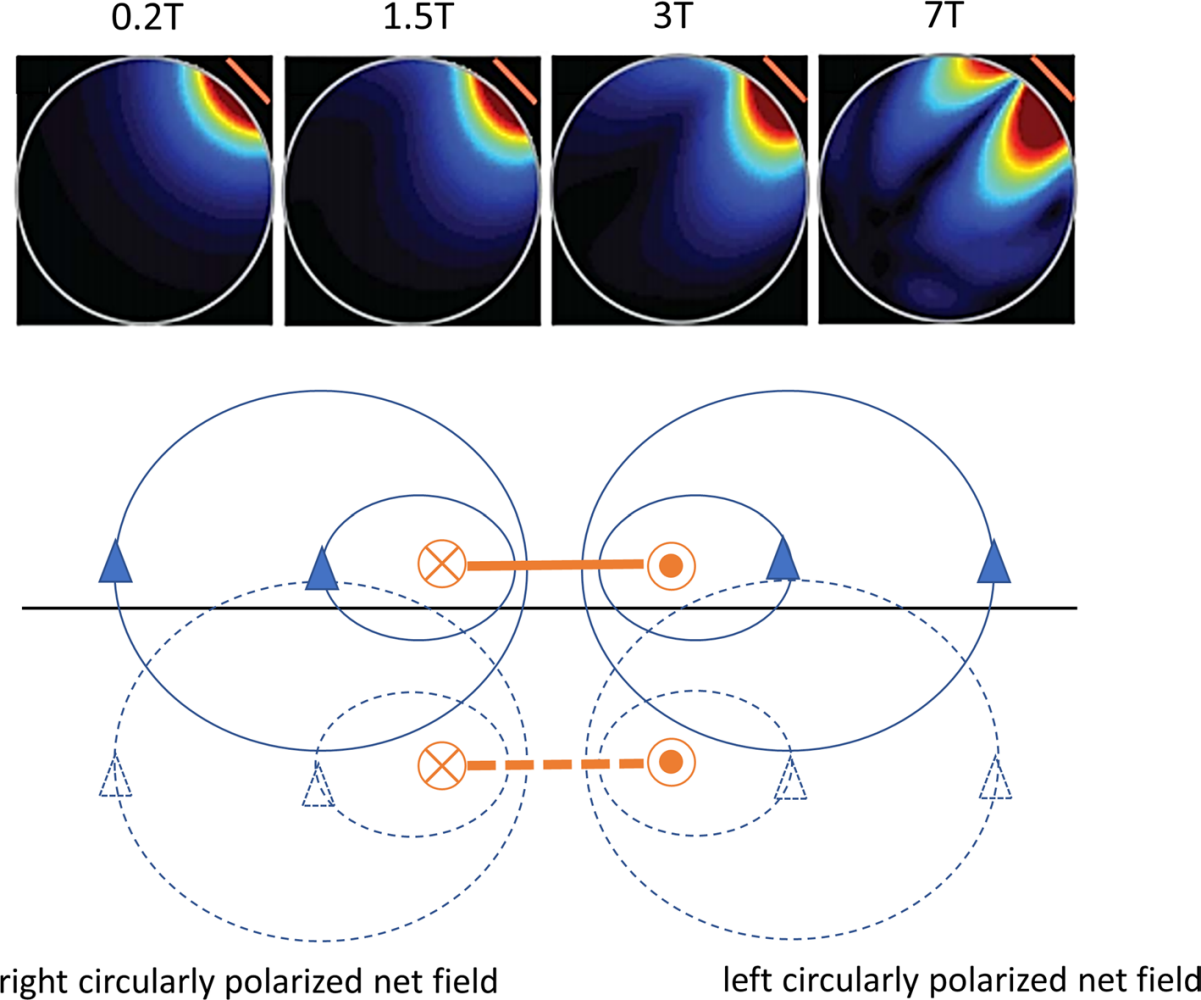
(Top) Simulated B_1_^+^ fields produced by a surface coil as a function of field strength. With increased field there is an increase in spatial inhomogeneity. B_1_^−^ fields are the mirror images of these fields. (Bottom) Mechanism by which asymmetries in B_1_^+^ or B_1_^−^ are produced in a conducting sample. The RF surface coil is denoted in side-view by the solid orange line. The magnetic field produced is shown in solid blue. This time-varying magnetic field produces eddy currents within a conducting sample: the spatial distribution of these eddy currents can be calculated using the concept of a mirror conductor shown as the dotted orange line, which produces mirror magnetic fields shown as dotted blue lines. A key element is that the fields from the coil and its mirror image are out-of-phase with one another. By considering the superposition of main and mirror magnetic fields, right circular polarization (contributing to B_1_^+^) or left circular polarization (contributing to B_1_^−^) dominate on the two respective sides of the coil. Figure adapted from Ref. [11]

The spatial inhomogeneity in the B_1_^+^ field is due to the effects of the permittivity and conductivity of the sample [10]. With respect to the permittivity, since the wavelength (*λ*) of the transmitted EM as it passes through the body is inversely proportional to the square root of the permittivity, Eq. (1), there are increased “wave effects” where this phenomenon essentially corresponds to constructive and destructive interference patterns within the sample.

1 $$\lambda \sim \frac{c}{{f\sqrt {\varepsilon_{0} \varepsilon_{{\text{r}}} } }}$$

where *ε*_0_*ε*_r_ is the permittivity of the tissue, *f* the MRI operating frequency and *c* the speed of light. The effect of tissue conductivity is to attenuate the EM field due to an effective skin-depth (*δ*), Eq. (2).

2 $$\delta \sim \sqrt {\frac{1}{{\pi f\mu_{0} \sigma }}}$$

where *μ*_0_*μ*_r_ is the permeability of tissue. The image of course contains contributions both from the transmit B_1_^+^ and also receive B_1_^−^ EM fields. In its simplest form, for a simple three-dimensional (or 2d with ideal pulse profile) low-tip angle gradient echo sequence, ignoring any effects of T_1_ and T_2_* relaxation, the signal intensity is proportional to:

3 $$S \propto \left[ {\sin \gamma B_{1}^{ + } \tau } \right]\left( {B_{1}^{ - } } \right)^{*}$$

where the first term represents the excitation tip angle, and the second term the complex conjugate of the receive field. For the vast majority of clinical scans, the receive coils are loops which are part of a larger receive array. At low fields (1.5 T and 3 T) the reception field of a loop is symmetric with respect to the axis of the loop, but at high field the pattern become asymmetric, as shown in Fig. 2 due to eddy currents [11].

The other parameter which is strongly affected by interactions of the EM energy with the body is the specific absorption ratio (SAR). In simple terms the SAR is expected to increase as the square of the magnetic field up to at least 7 T. However, the spatial inhomogeneity of both the magnetic and electric field components of the transmitted power means that the situation is more complicated.

Since the B_1_^+^ field is spatially inhomogeneous, the amount of power required for a particular imaging sequence using pre-defined tip angles depends upon the region being studied. For example, for 7 T imaging or spectroscopy of the temporal lobe using a standard quadrature transmit coil, the amount of transmit power required to reach a given tip angle is much higher than if the hippocampus were to be the target. As a result, the SAR of the scan would increase significantly. We also need to consider the fact that, despite the transmit coil and the subject being transversely symmetrical, the SAR has a non-symmetric distribution.

So overall, the situation is that if a circularly (or elliptically) polarized transmit coil is used for neuroimaging at 7 T or body imaging at 3 T there is a fixed B_1_^+^ distribution which is perfectly adequate for imaging, for example, the hippocampus at 7 T or the liver of patients with average body mass indices (BMIs) at 3 T, but is sub-optimal for cases requiring either: (i) a homogeneous transmit field throughout the subject, or (ii) a high transmit efficiency in a region where the subject geometry means that the efficiency is intrinsically low. In such cases, one would like to have a way to manipulate the field distribution.

The most obvious approach, as mentioned in the introduction, is to use a parallel transmit approach, using a multi-element transmit array in which the magnitude and the phase of the RF transmitted from each element can be individually controlled. Indeed the first transmit arrays designed for 7 T neuroimaging were described by the Minnesota group in 2005 [4], and the first experimental eight-channel transmit array for body imaging in 2007 [12–14]. Multi-transmit technology is present on all modern-day 7 T scanners (the first commercial dual-transmit 3 T systems were introduced in 2010 [15, 16] and this technology has also become the de facto state-of-the-art). Eight-element transmit head coils are now readily available commercially, as are multi-element dipole-based systems for body imaging [17–20]. However, this represents the situation in the research environment. As of 2021 there are almost no clinical studies being carried out in parallel transmit mode, primarily due to the fact that commercial systems are currently only FDA/CE approved for single transmit mode operation. Even in “research mode” these systems have such restrictive safety limits, that the full advantage of parallel transmit technology has not yet been reached.

An alternative approach, which was developed initially as a “stop-gap” measure at 7 T to improve image quality while the more complicated and expensive parallel transmit technology was being developed, is to use specialized high permittivity materials (HPMs), also termed “dielectric pads” which are placed around the head or body to tailor the RF distribution. The following section gives a short summary of initial results obtained at many sites using this approach.

## Initial results obtained at 7 T and 3 T using metal titanate aqueous suspensions

If an HPM is placed inside the transmit coil, when an RF pulse is transmitted the oscillating electric field from the transmit coil induces displacement currents in the HPM, which in turn produce a secondary magnetic field *B*_sec_ described in Eq. (4):

4 $$\nabla \times B_{\sec } = \mu \left( {J_{{\text{c}}} + J_{{\text{d}}} } \right) = \mu \left( {\sigma E + j\omega \varepsilon_{0} \varepsilon_{{\text{r}}} E} \right)$$

The total current consists of two components, a conductive current *J*_c_ and a dielectric displacement current *J*_d_. The *B* field produced by the dielectric displacement current is approximately proportional to frequency and the relative permittivity of the HPM. To first order, the effect depends upon the product of the permittivity and thickness of the HPM. Initial results showing altered distributions of the RF field were reported by Alsop [5] at 4 T and Yang at 7 T [6] using large bags of water placed around the head. Similar results were shown for abdominal imaging at 3 T using even larger gel-based pads [21, 22].

In practise there is very little space inside a head coil at 7 T, and so to maintain the full versatility of the close-fitting multi-channel receive array in terms of parallel imaging performance the HPM needed to be made much thinner and also relatively flexible. The first work to show a practical approach for improving the image homogeneity for in vivo 7 T imaging was by Haines et al. [23] who designed thin (< 1 cm) pads containing a slurry of calcium titanate (CaTiO_3_) in either H_2_O or D_2_O with relative permittivities up to 110–130 (depending on the particle size). These slurries are physicochemically stable and have very short T_2_ and T_2_* water relaxation times. These “dielectric pads” now form the basis of commercial products (https://www.multiwaveimaging.com), and have been used in a large number of studies.

**Fig. 3:**
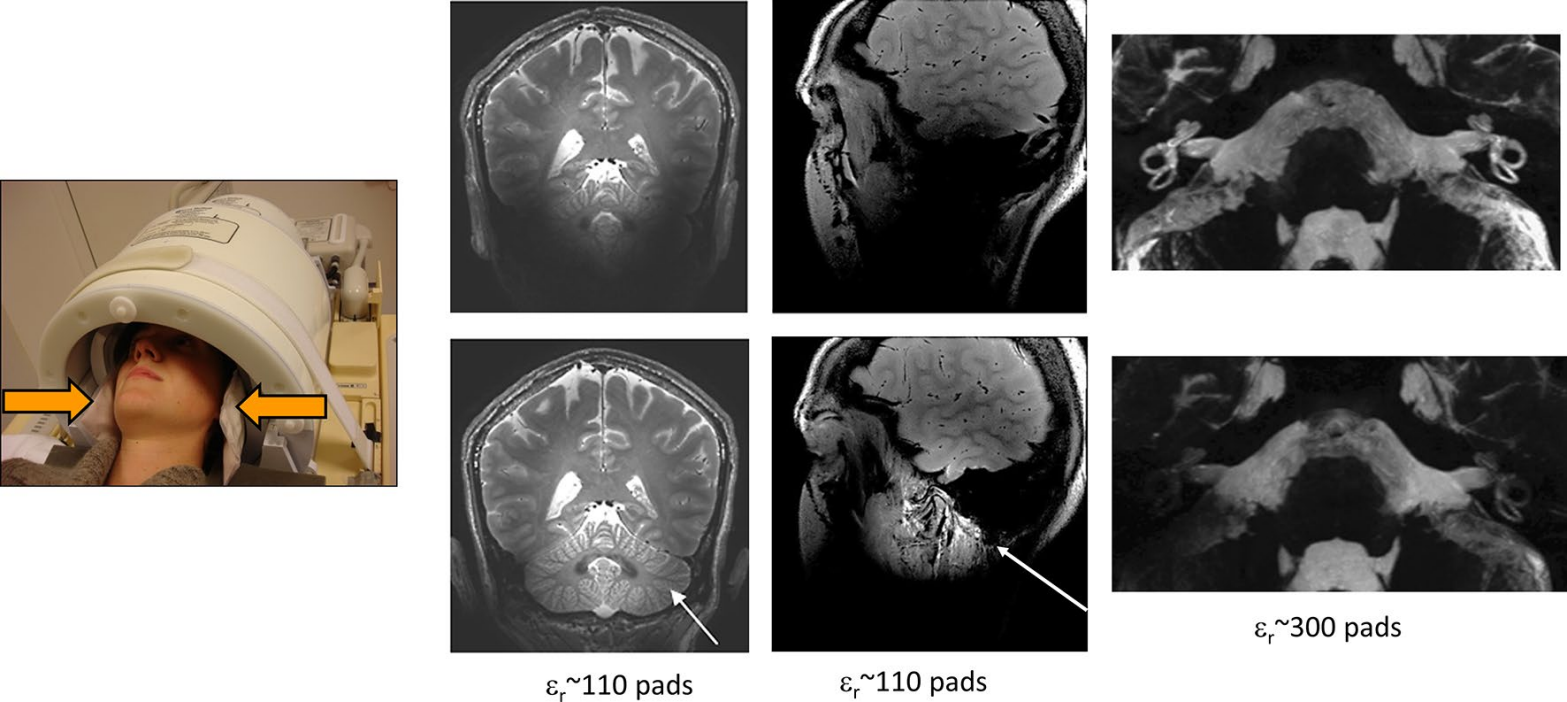
Placement of pads within a 32-channel receive coil array for neurological applications at 7 T. (Left) T_1_-weighted images acquired with and without calcium titanate pads in place (arrow indicates area of greatest signal increase) [29]. (Centre) Images of the temporomandibular joint (TMJ) with and without calcium titanate pads [27] (right). Images with and without barium titanate pads of the inner ear of a healthy volunteer [24]

While carrying out various simulations, it became clear that higher values of permittivity could be used to target the B_1_^+^ field to specific regions of the brain: this proved to be very useful for applications such as inner ear [24–26] and temporomandibular joint [27]. Fairly quickly other metal titanate formulations, BaTiO_3_ slurries [8] and BaTiO_3_ sintered beads [28], with much higher permittivities (> 300) were investigated with the aim of targeting a strong increase in transmit efficiency to specific places within the brain [24–26]. Figure 3 shows examples of applications of these two (homogenizing and targeting) approaches.

**Fig. 4:**
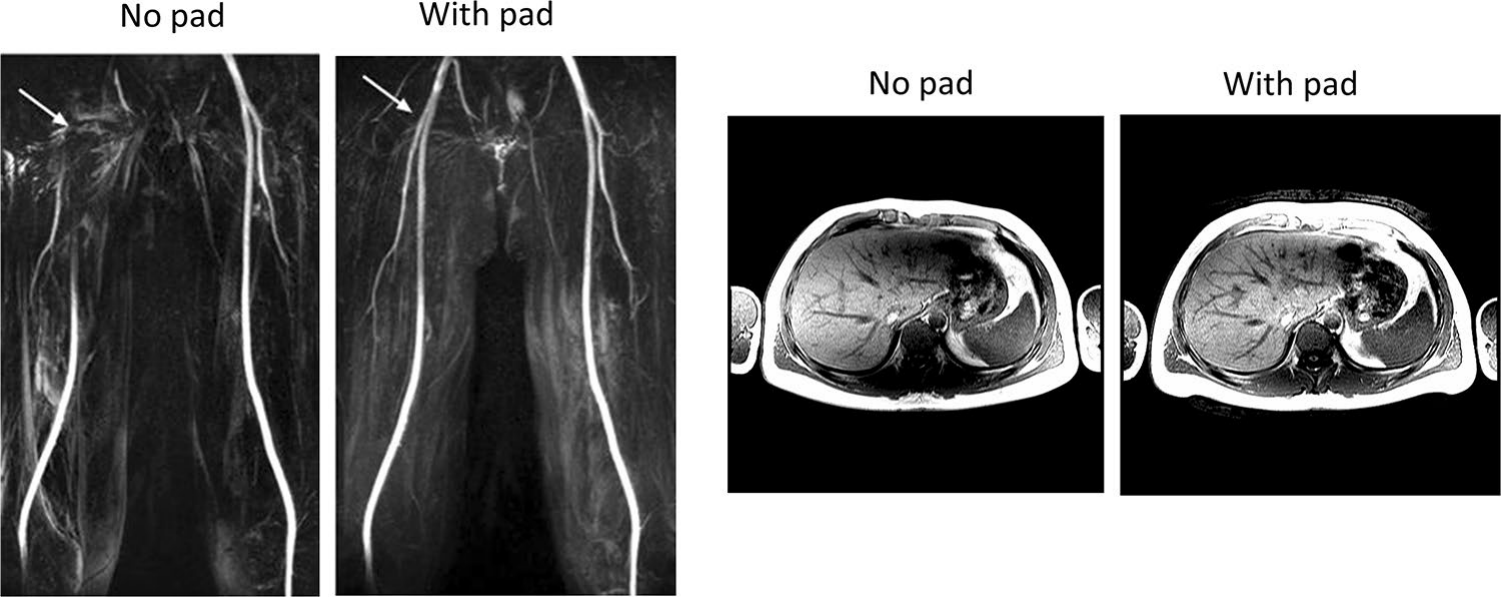
(Left) Representative maximum intensity projections from a volunteer with no dielectric pad in place and a 2 cm thick barium titanate high-permittivity pad. The dielectric pad significantly increased signal around the bifurcation point of the common femoral artery (arrow). Figure reproduced from [32]. (Right) Images acquired through the abdomen of a healthy volunteer with and without a 2 cm thick barium titanate pad. Figure reproduced from [9]

HPMs can also be used at 3 T, where image-shading is in general lower but can be especially troublesome in body and cardiac imaging. Equation (4) shows the secondary magnetic fields produced by an HPM are inversely proportional to frequency, and so materials used at 3 T must have high permittivity values. Brink et al. performed a number of studies using barium titanate-based pads for 3 T cardiac imaging using both single-transmit and dual-transmit commercial systems, and showed improvements in transmit efficiency and image contrast homogeneity [30, 31], as well as reduced SAR. Similar results were shown in general abdominal imaging [9]. Other groups picked up on these ideas and designed HPM slurries for their own particular applications such as imaging of the femoral arteries [32]. Some examples are shown in Fig. 4.

## Development of new ceramics as high permittivity materials for MRI

**Fig. 5:**
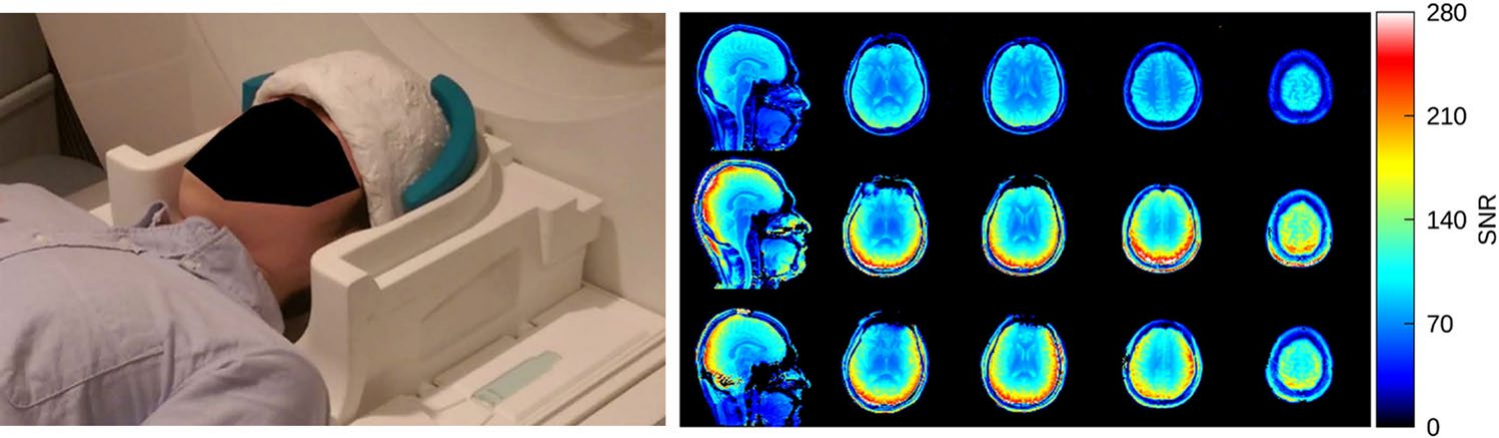
(Left) Photograph of a moulded high permittivity ceramic helmet surrounding the head on a 3 T scanner. (Right) SNR maps from a subject, acquired with a 20-channel receive array head coil (top row), 64-channel receive array head coil (middle row), and 20-channel receive array coil with ceramic helmet (bottom row). The SNR maps were normalized by the corresponding acquired B_1_^+^ map. Figures reproduced from Ref. [34]

Although the permittivity of barium titanate based HPMs can be increased by ~ 10–40% by forming sintered beads [28] or by compression [33], to achieve the very high permittivities required for optimal operation at clinical field strengths it was clear that materials with a high density would be required. Mike Lanagan’s group at the Materials Research Institute at Penn State University in the USA had extensive experience of high permittivity ceramic design, and together with the MRI group of Qing Yang and Chris Collins, Sica et al. [34] designed a helmet for neuroimaging at 3 T, constructed from materials with either a permittivity of 1000 or 1200, both materials having a low conductivity of 0.05 S/m. They obtained images which showed similar signal to noise ratio (SNR) maps using a 20-channel receive coil with the HPM helmet to those obtained using a 64-channel coil without the HPM insert, as shown in Fig. 5 (this helmet approach has subsequently been used for neuroimaging at 7 T and 10.5 T with lower permittivity materials [35, 36]). One of the questions which often arises is how can the SNR be increased when typically body loss dominates over coil loss at clinical field strengths? Vaidya et al. [37] provided an explanation based on analyzing the RF transmit and receive field by combining a surface loop coil with an HPM. They concluded that the effect of the HPM is to increase the effective number of receive coils, which would more closely approach the ultimate intrinsic SNR [38, 39].

**Fig. 6:**
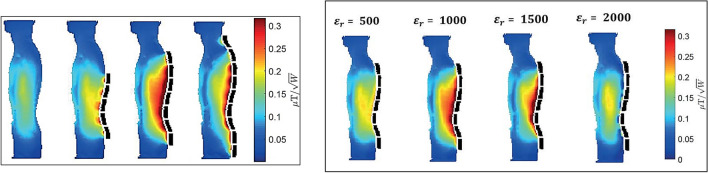
(Left) Interaction of different numbers of PZT blocks (*ε*_r_ ~ 1000) with a quadrature excitation RF field at 3 T. The increase in transmit efficiency varies with the number of PZT blocks (optimum number seven). (Right) The effect of different permittivities of the seven PZT blocks on the transmit field. Conductivity values were kept constant and equal to 0.38 S/m. The transmit field has an optimal efficiency between *ε*_r_ ~ 900 and *ε*_r_ ~ 1,300. Figure adapted from Ref. [40]

Following the lead from the Penn State researchers, Koolstra et al. [40] used lead zirconate titanate (PZT) blocks with *ε*_r_ ~ 1000, to investigate potential improvements in image quality and power requirements of spine imaging on a dual-transmit 3 T system. An optimal configuration was found via EM simulations, and the effect of the one-dimensional array of blocks on the transmit efficiency, receive sensitivity, SNR, power deposition, and clinical image quality was analyzed. One of the novel aspects of this work was that many individual blocks were connected together via conducting copper strips to form a much larger effective dielectric than could be produced in one piece. Simulation results showed that the PZT blocks improved the transmit efficiency by 75% while the global maximum SAR was decreased by 20% for the model Duke. In vivo experiments in ten healthy volunteers show statistically significant improvements for the transmit efficiency and receive sensitivity. The required system input power was reduced and distributed more equally over the transmit channels compared to active RF shimming. The blocks improved the image quality significantly, but did lead to over-tipping in some female volunteers (Fig. 6).

**Fig. 7:**
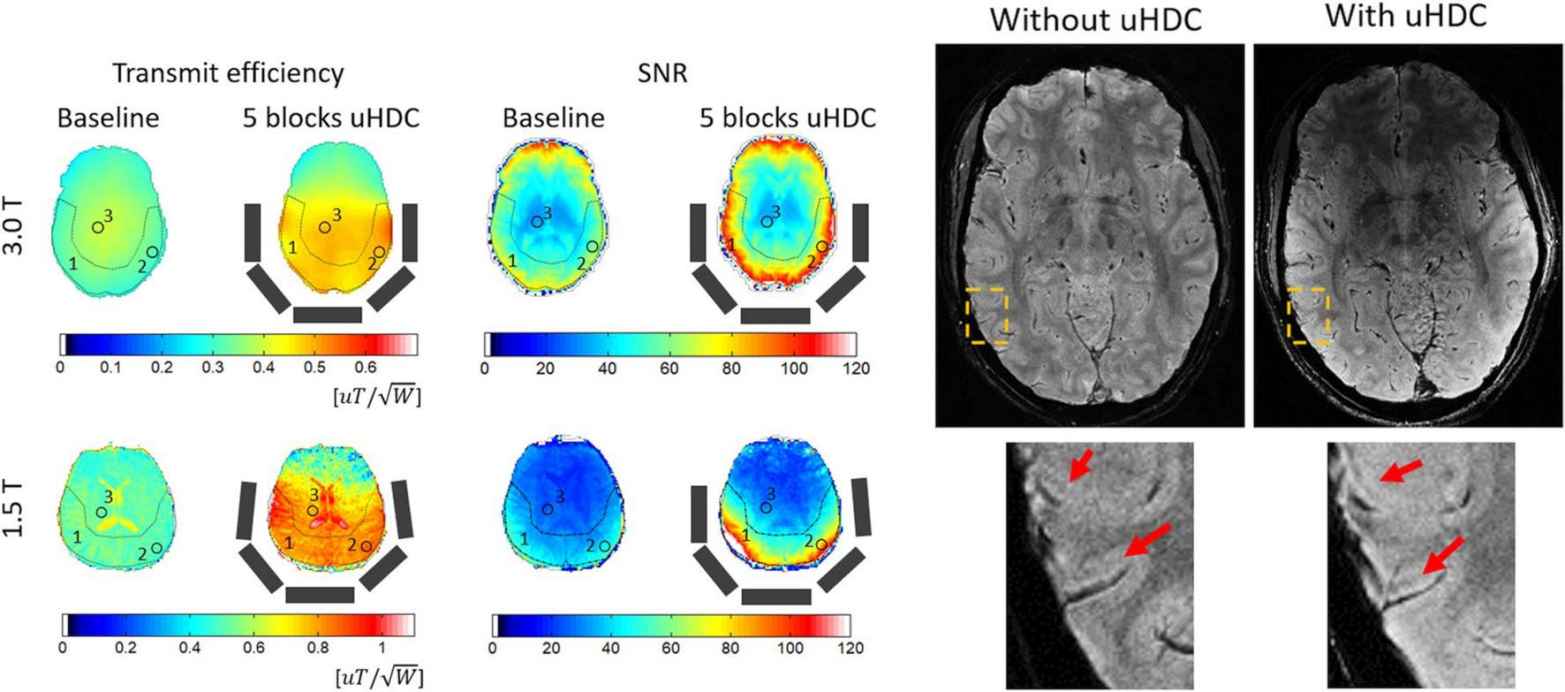
(Left) Experimental B_1_^+^ efficiency and SNR maps from a human brain (skull and CSF stripped) at 3 T and 1.5 T without and with five blocks of HPM drawn as grey rectangles. (Right) High-resolution (0.31 × 0.31 × 0.75 mm^3^) T_2_* gradient-echo images of the brain acquired at 3 T without and with HPM blocks. With the improved SNR with the HPM the layer structures in the cortex can be seen more clearly (arrows), while RF transmission power was reduced by 45%. Figures reproduced from [41]

The next major step in material design with even higher permittivities came again from the Lanagan group at Penn State. Rupprecht et al. [41] designed specialized ceramics based on Pb(Zr_x_Ti_1-x_)O_3_ at the morphotropic phase boundary to produce materials with *ε*_r_ > 3000 for both 3 T and, for the first time, 1.5 T applications. The authors showed significant increases in the local transmit efficiency and SNR for both 3 T and 1.5 T neuroimaging, as depicted in Fig. 7.

**Fig. 8:**
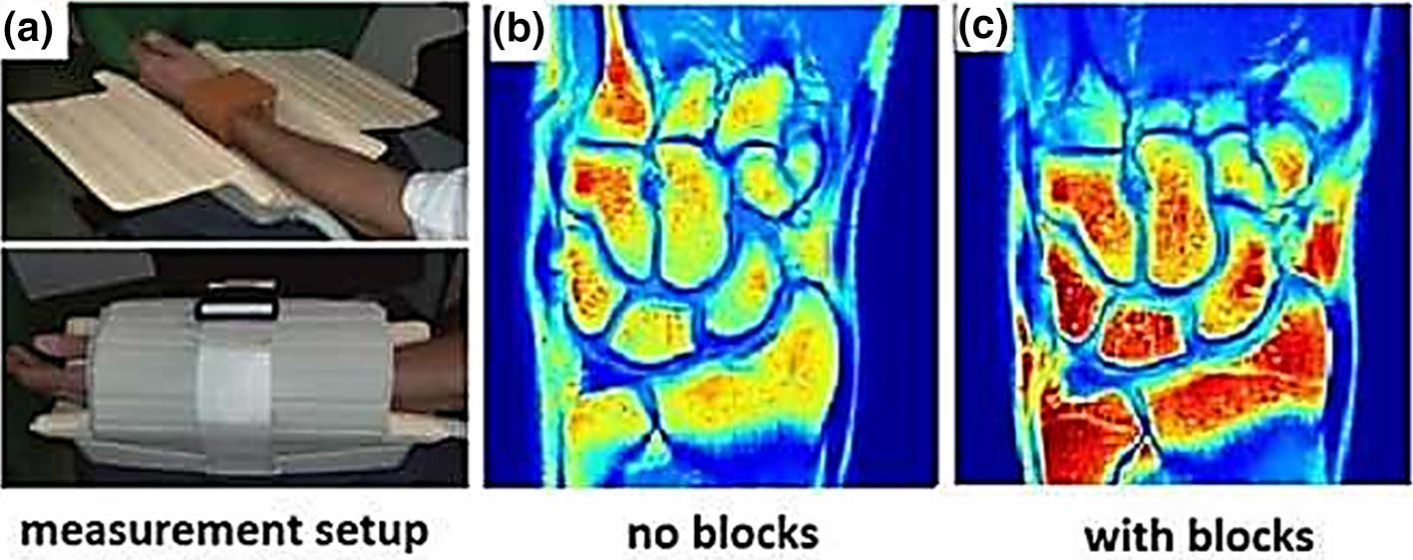
**a** Photographs of an in-vivo setup at 1.5 T using a commercial eight-channel receive array coil with ceramic blocks (*ε*_r_ ~ 4500) placed around the wrist of a healthy volunteer. **b** T_1_-weighted wrist images without blocks, and **c** with blocks. The SNR is approximately 45% higher with the blocks over an ROI comprising the entire imaged volume. The power level from the transmitter coil was 40% lower with blocks compared to without blocks. Figure reproduced from Ref. [42]

Working in a new collaboration with the School of Physics and Engineering at ITMO University in St. Petersburg, classes of material based on BaTiO_3_ (with ZrO_2_ and CeO_2_-additives) with an *ε*_r_ ~ 4500 were developed in Russia and tested by Irena Zivkovic in Leiden for scanning extremities at 1.5 T [42]. Adding cerium and zirconium oxides shifts the Curie temperature and blurs the phase transition. This material exists in a paraelectric phase, i.e., it is a ferroelectric with a Curie point below room/body (operating) temperature with a spontaneous dielectric polarization below its Curie temperature. Figure 8 shows that for in vivo wrist experiments, the SNR of a commercial eight-channel receive array was improved by ∼45% with the ceramic.

**Fig. 9:**
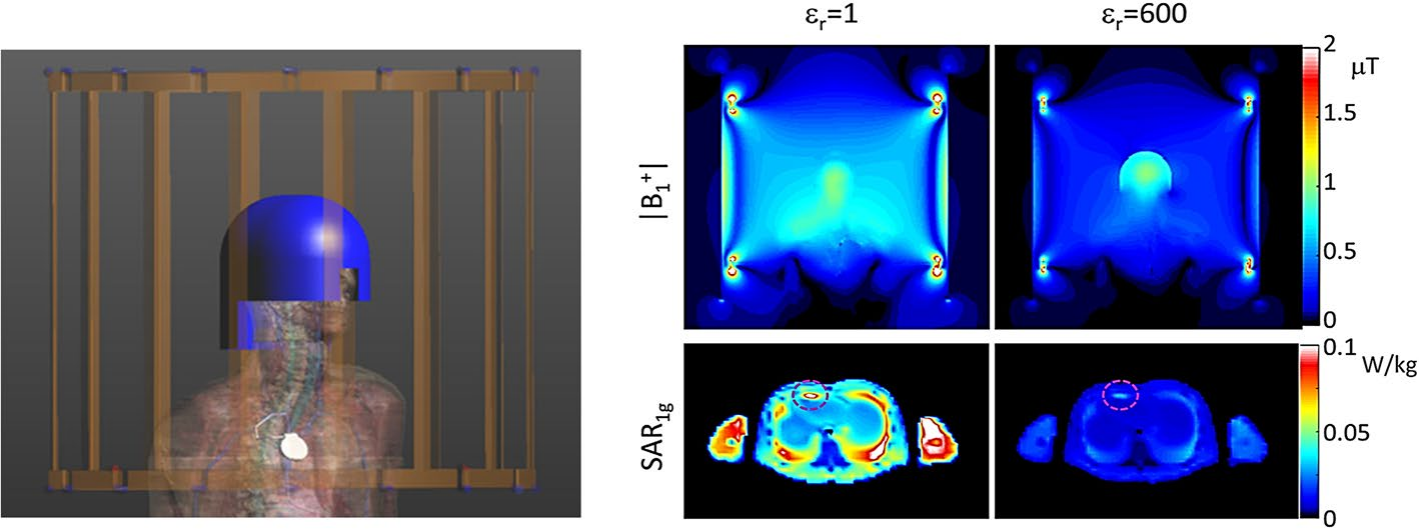
Simulated illustration of the use of HPMs for neuroimaging in a patient with a cardiac pacemaker. (Left) Numerical model of the 3 T body coil (gold) loaded with a human model with high-permittivity helmet (blue) and pacemaker with implanted lead (silver). (Right) The B_1_^+^ field and SAR_1g_ for an excitation with the body coil driven to produce 1 mT at the centre of brain without or with a 5-mm-thick helmet (*ε*_r_ = 600) Figure reproduced from [43]

It is worth noting that while increases in SNR using HPMs are potentially very useful clinically, there is another general class of scenarios in which HPMs might be equally useful, as pointed out in a publication from New York University [43]. These are based on the ability of an HPM to increase the local transmit field. The clinical situation is one in which a patient has a medical implant which, due to the danger of heating caused by the implant, means that a “reduced power” imaging protocol must be run, which generally results in sub-optimal image quality due to required changes in imaging parameters such as refocusing angle, repetition time, minimum echo time and echo train length. However, very often the imaging region-of-interest (ROI) is physically different from the location of the implanted medical implant, for example a patient with a cardiac stent who requires imaging of a specific section of the spinal column or a knee scan. In this case, by placing the HPM next to the imaging ROI to increase the local transmit efficiency the scan can be run with all imaging parameters identical to the optimal scan, except with a lower RF transmit power, resulting in normal image quality. EM simulations performed by Yu et al. [43] at 3 T, in which HPMs were placed around the imaging ROI to concentrate the local transmit field produced by the body coil, resulting in a reduction in the required power transmitted by the body coil for a given image contrast, and an associated reduction in the SAR averaged over 1 g of tissue next to a pacemaker lead by almost 75% [43]. This approach, shown in Fig. 9, could also potentially be combined with other promising approaches for imaging patients with implants such as parallel RF transmission [44, 45], or using coil geometries which are specifically designed to reduce the electric fields in pre-determined areas [46, 47].

## Software tools to design the geometry and properties of HPMs

**Fig. 10:**
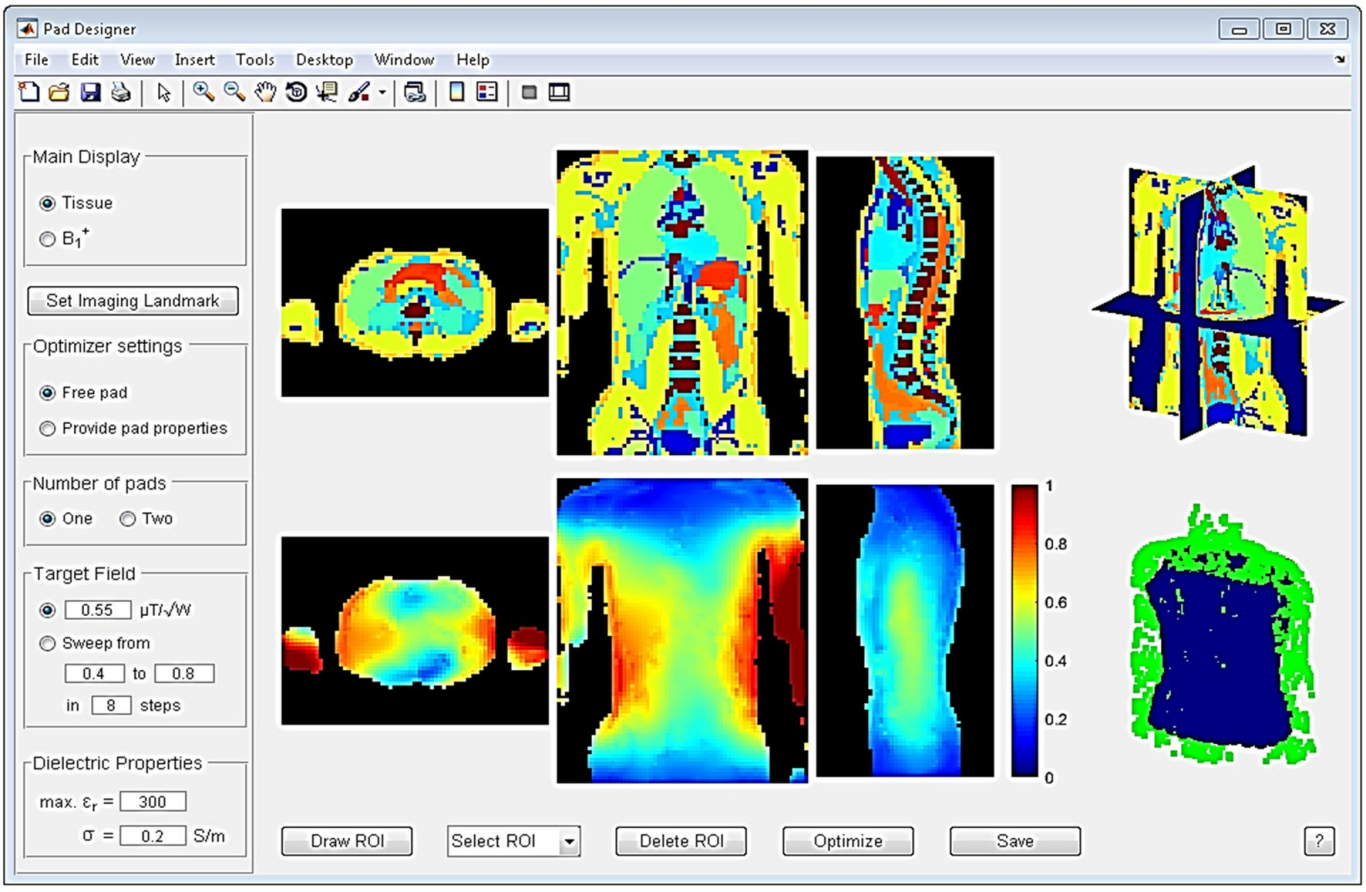
Graphical user interface of the dielectric pad design tool. Tissue profiles are shown in the top row, with B_1_^+^ fields in the bottom row. After a ROI is drawn, the user can start the optimization with the selected options. Figure reproduced from Ref. [48]

Despite the relative ease of physically constructing dielectric pads or ceramic blocks, their optimal design, in terms of material properties and dimensions, is not trivial as these depends on many aspects; the optimal design varies with imaging region-of-interest, dimensions of the body, and MR configuration (e.g. static field strength and form of transmit/receive coils). Therefore, the dimensions, location, and constitution need be optimized in an application-specific manner. One common approach is to perform a parametric optimization using general-purpose electromagnetic field solvers, based on a systematic trial-and-error approach and guided by user intuition, and then to choose the best pad-properties afterwards. As each of these simulations involve a large computational domain with an RF coil and heterogeneous body model, such procedures typically take multiple days for a single application. This limits the exploitation of this practical shimming approach. Several methods have been developed to accelerate this process, but unless these are made readily available the chances of other researchers coming up with new applications are very low. To enable the use of HPMs to be more widespread, van Gemert developed an easy-to-use software tool [48] which allows researchers and clinicians to design dielectric pads for 7 T neuroimaging and 3 T body imaging applications on standard PC systems. The tool incorporates advanced computational methods based on field decomposition and model order reduction [49, 50] as a framework to efficiently evaluate the B_1_^+^ fields resulting from dielectric pads. The interface is shown in Fig. 10.

**Fig. 11:**
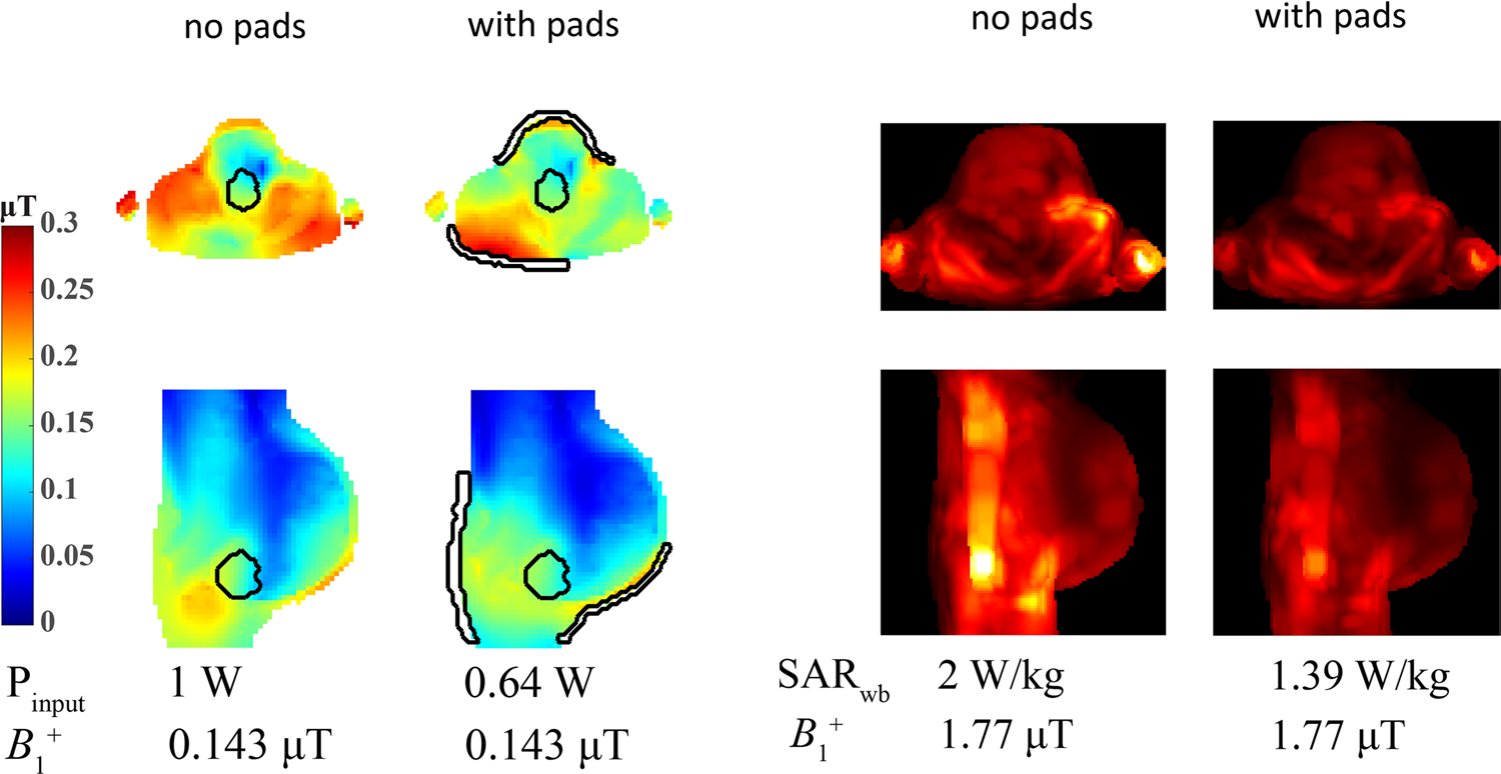
Results from a simulation study on the effects of an optimized dielectric pad (in this example optimized over the volume of the fetal brain) on pregnant body models. (Left) B_1_^+^ fields for the ninth month of gestation. The cross-sections shown are obtained through the midpoint of the ROI normalized to 1 W input power. For an equal B_1_^+^ magnitude within the ROI the input power is reduced significantly. (Right) Corresponding SAR plots which show a reduced SAR associated with reaching a given B_1_^+^ transmit field. Figures adapted from Ref. [51]

One example of using such a tool is shown in Fig. 11, which represents a simulation-only study performed to determine the potential of using HPMs in improving image quality for fetuses at different stages of development. Pregnant models in the third, seventh, and ninth months of gestation were used for the simulations at 3 T. Dielectric pads were optimized for two ROIs: the entire fetus and the brain of the fetus. The SAR distribution was evaluated in terms of the whole-body SAR, average SAR in the fetus and amniotic fluid, and maximum 10 g-averaged SAR in the mother, fetus and amniotic fluid. Results showed that the optimized dielectric pads increased the transmit efficiency by up to 55% and increased the B_1_^+^ homogeneity in almost every tested configuration. The whole-body SAR was reduced by more than 31% for all body models.

As seen in the previous sections, highly specialized ceramic materials are needed to generate the high values of permittivity necessary for applications at clinical field strengths. Such materials can be quite heavy, and they can take up significant space in close-fitting receive arrays. In collaboration with researchers in St. Petersburg, we developed a more flexible approach based on passive devices which are often referred to in the literature as artificial dielectrics, metasurfaces, metamaterials or metamaterial-inspired structures. The development of these types of materials is summarized in the next section.

## Design of metamaterials/metasurfaces and artificial dielectrics to enhance image quality

The term “metamaterial”, also referred to as a “left-handed medium” was originally associated with a medium which had both negative permittivity and negative permeability in the same frequency range. Wave propagation in such materials was theoretically analyzed by Veselago in his seminal paper in the late 1960s [52]. It took more than 30 years to implement these and related materials [53–56]. Subsequently, the term “metamaterials” was expanded to comprise artificial composite materials, typically constructed from sub-units which are sub-wavelength in dimensions, that exhibit electromagnetic properties which cannot be found in naturally available materials. Most applications of metamaterials have been demonstrated in the optical (tens to hundreds of THz) and microwave (tens to hundreds of GHz) frequency ranges using sub-millimeter-sized unit-cells, although there have also been applications in the low MHz range [57] and also for acoustics [58, 59].

Despite there being quite a number of MRI-related papers with the term “metamaterials” in the title, there is still quite some controversy about whether these actually qualify as metamaterials, and indeed what the difference is between standard RF coils and metamaterials. One of the features of metamaterials is that they are constructed of sub-units which are sub-wavelength in dimensions. For MRI of course, ALL RF coils correspond to units, or contain sub-units, which are intrinsically sub-wavelength in dimensions. Perhaps the key point is that the function of a metamaterial is linked to the interactions between a number of identical (or similar) sub-wavelength sub-units, i.e. a metamaterial consists of a large number of highly coupled structures. So one can argue that the ubiquitous multi-mode birdcage coil is in fact a metamaterial!

**Fig. 12:**
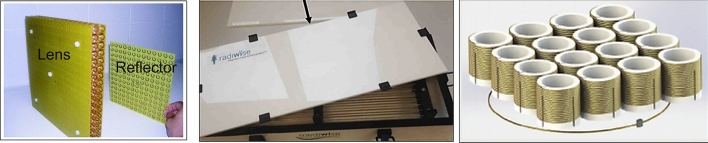
Photographs of various metamaterial-based prototypes for MRI. (Left) A metamaterial lens based on split rings [61], (centre) a structure based on conductive rods within a high permittivity liquid [67], and (right) the first metamaterial MRI structure published based on “swiss rolls” [65]

Historically, several studies showed proof-of-principle implementations of metamaterials in MRI using lenses based on split rings [60–64], Swiss-rolls [65], and discrete wires [66]. Metamaterials have mostly been forming resonant structures which are inductively coupled to the body transmit coil. Slobozhanyuk et al. used two layers of 14 metallic rods immersed in water to enhance the local magnetic field [67], and this basic structure was used in many follow-up experiments [68–70]. Similar devices have been formed by different research groups [71, 72]. However, since the size of the unit-cells for metamaterials for RF applications lies in the centimetre to tens-of-centimetre range (see Fig. 12), these 3D metamaterial structures are very large and cannot be placed inside a receiver coil array, meaning that there are currently no practical implementations of metamaterials on commercial MRI scanners.

**Fig. 13:**
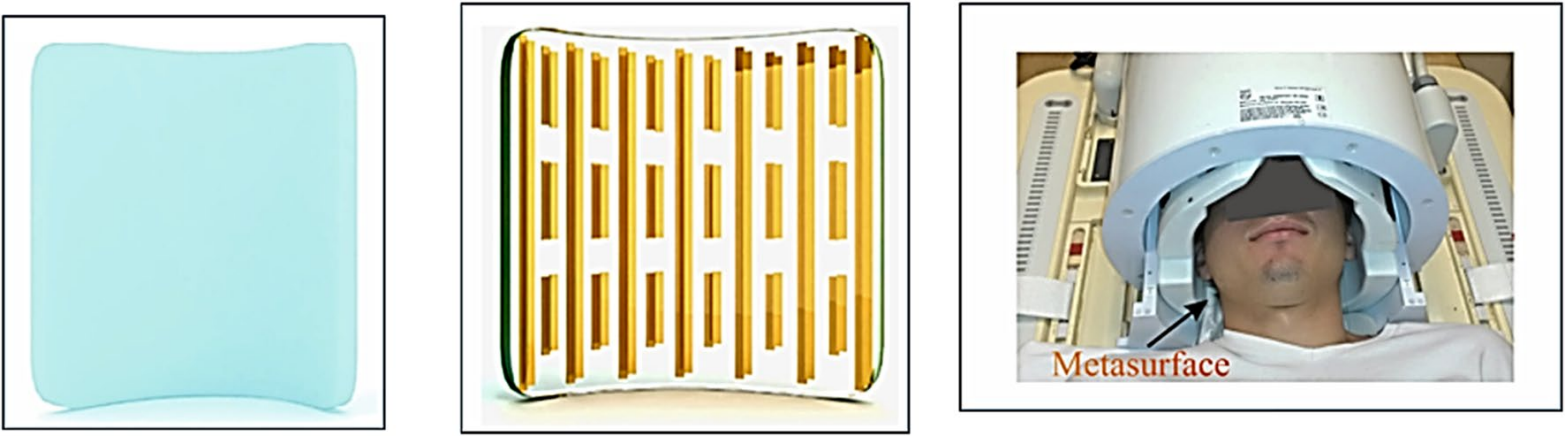
Combination of a high permittivity pad (left) with a wire-based pattern placed on top (centre) results in a thin metasurface which can be placed in between the subject and a close-fitting 32-channel receive array (right). Figure reproduced from Ref. [73]

From a practical point-of-view a structure which would fit inside existing receiver coil arrays is necessary to produce meaningful results. Schmidt et al. designed a two-dimensional metasurface [73] by combining a long/short wire design with a dielectric pad to produce a metasurface which can be place inside a commercial 7 T 32-channel receive array, as shown in Fig. 13. The length of the longer strips is designed to be slightly shorter than that required to produce the first half-wavelength resonance at 298 MHz. The length and the spacing between the strips (in combination with the dielectric substrate) control the frequency of the generated resonant modes [66, 67, 74]. These resonant modes are produced by the effective negative permittivity generated by the set of long strips. Incorporating a matrix of shorter strips gives additional flexibility in terms of shaping the local magnetic field enhancement. In the original publication [75] the short wire pairs represent an artificial “magnetic atom” which displays magnetic resonance [76]: in contrast, in our design the non-resonant short strips are used to modify the near field pattern of the eigenmode to obtain a greater increase in the MRI sensitivity and to perform fine tuning of the metasurface.

**Fig. 14:**
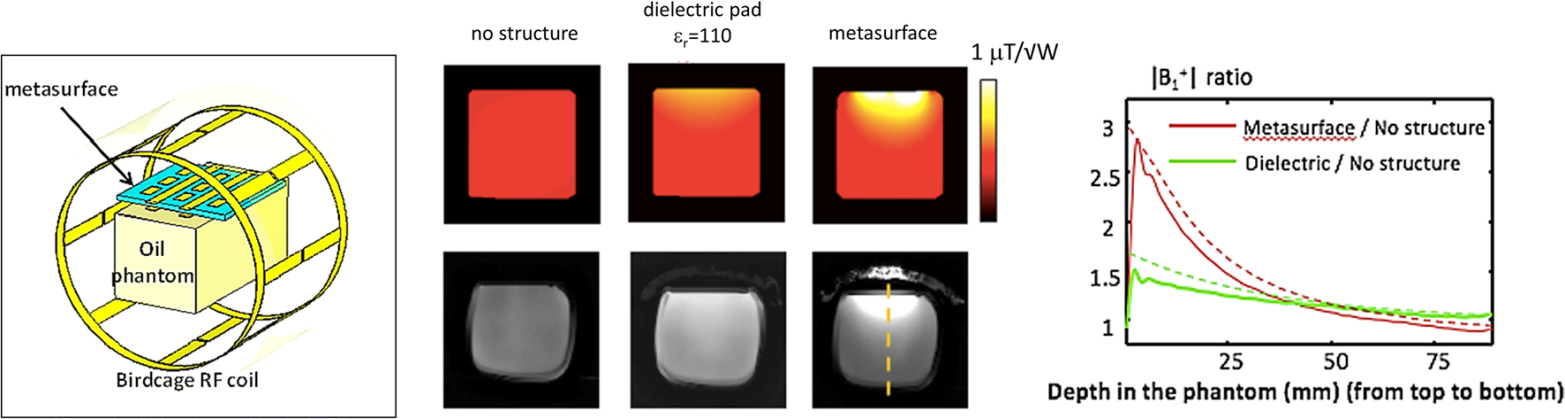
(Left) Setup for simulations and experimental testing of the metasurface. (Centre). Simulated B_1_^+^ field (top) and corresponding low tip angle gradient echo images. (Right) Measured (solid line) and simulated (dashed line) B_1_^+^ profile, along the yellow dashed line. Figure reproduced from Ref. [73]

Figure 14 shows that in simulations and corresponding experimental results, the integration of a dielectric pad into a metasurface effectively mimics the behaviour of a much thicker pad (or a much higher dielectric constant).

**Fig. 15:**
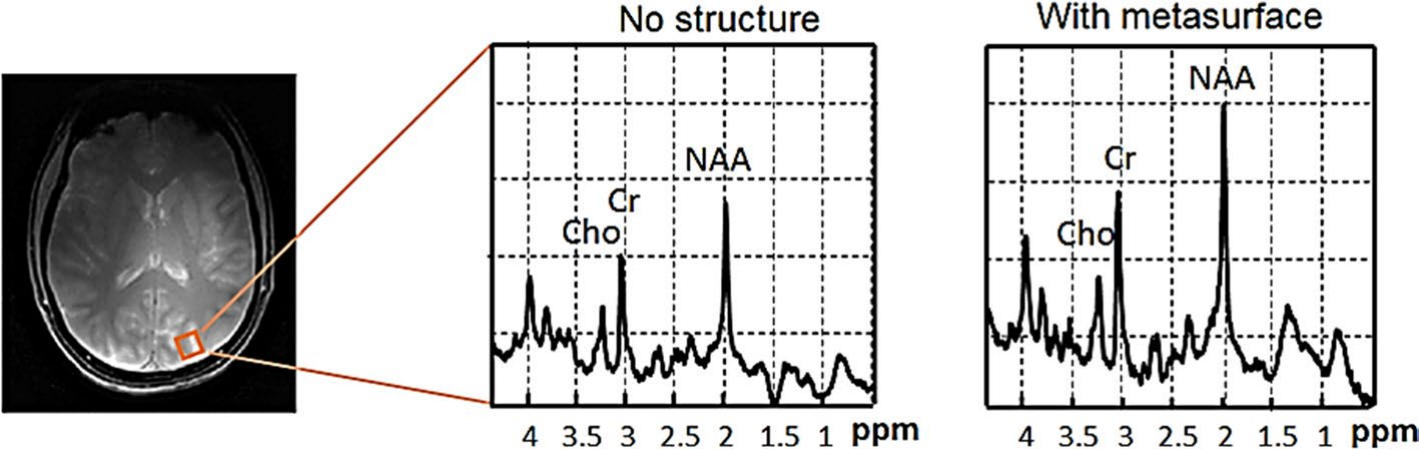
Localized ^1^H spectroscopy performed at 7 T without (left) and with (right) a metasurface in place (the location of the voxel is shown in the anatomic image). The intensity of the spectral plots is normalized to the root-mean-square noise. Figure reproduced from [73]

In vivo brain scanning was performed with the metasurface structure placed close to the occipital cortex. A quadrature birdcage coil was used for RF transmission, and a close-fitting 32-channel array coil for signal detection. Four volunteers were scanned and the average transmit efficiency over the ROI increased by 2.0 ± 0.3, the SAR efficiency increased by a factor of 1.6 ± 0.24, and the receive sensitivity by 1.9 ± 0.2. A localized 1H spectrum from a small voxel in the occipital lobe, taken with and without the metasurface in place, is shown in Fig. 15. An increase of 50% in the SNR of the spectra was obtained using the metasurface, which agrees well with the increase in the simulated B_1_^−^ field integrated over the spectroscopic volume.

**Fig. 16:**
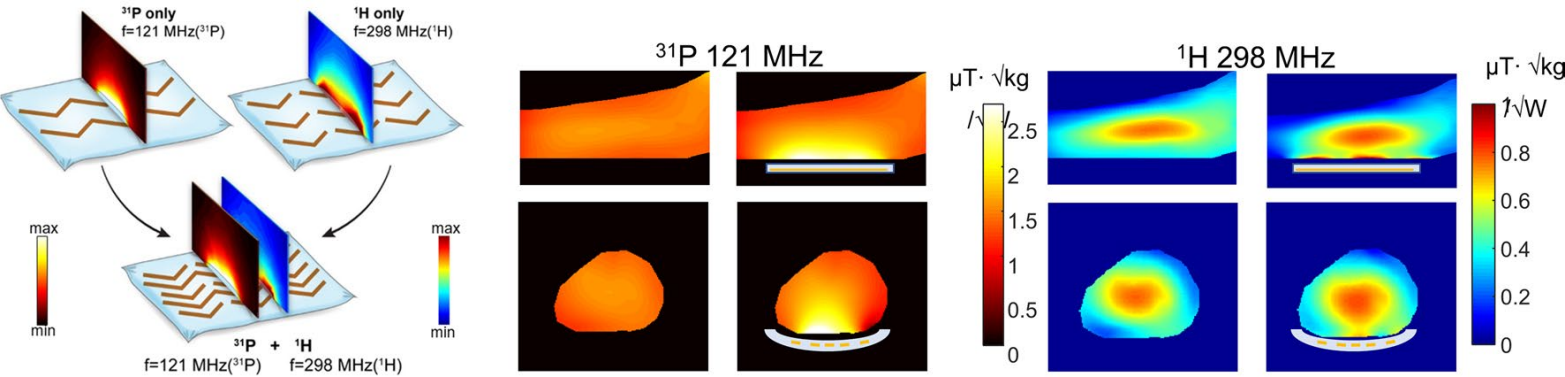
(Left) A schematic view of the metamaterial pad combined from ^1^H and ^31^P sub-setups. (Centre) Electromagnetic simulations at the ^31^P frequency with and without metamaterial, placed under the calf muscle. (Right) Electromagnetic simulations for ^1^H with and without metamaterial. Sagittal and axial cross sections through the calf are shown. The B_1_^+^ maps are normalized to the square root of maximum local SAR averaged over 10 g. Figures reproduced from Ref. [77]

Since one of the focuses of our high-field work in Leiden was in-vivo ^31^P spectroscopy of patients with Duchennes’ disease, we were interested to see if dual-tuned metasurfaces were feasible. Schmidt et al. [77] designed a new type of dual-nuclei resonant metasurface, which combines two configurations, one based on a set of electric dipoles for the low frequency band, and the other based on a set of magnetic dipoles for the high frequency band. A set of long strips produces resonant modes for the low frequency, and a set of short strips produces resonant modes for the high frequency. The two structures were harnessed together to generate a unique single metamaterial structure for dual-nuclei purposes as shown in Fig. 16, designed for both ^1^H (298 MHz) and ^31^P (121.7 MHz) frequencies at 7 T.

**Fig. 17:**
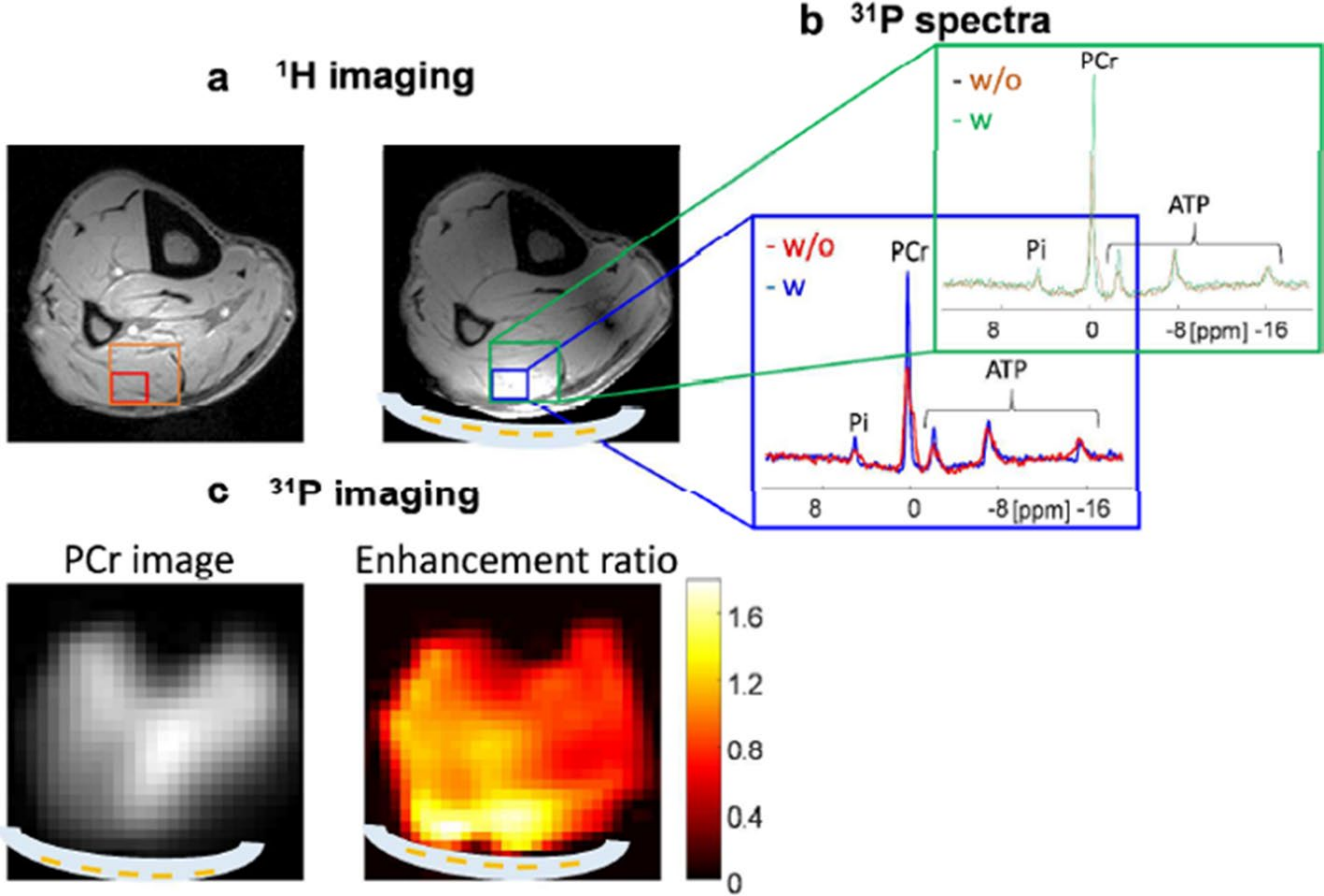
**a** ^1^H images at 7 T with and without the double-tuned metasurface in place. A double-tuned birdcage coil was used for transmit/receive. **b** ^31^P spectra from two different-sized voxels (the small voxel in blue corresponds to the volume of maximum signal enhancement, the larger voxel in green is the combination of the four voxels with the highest enhancement. **c** phosphocreatine (PCr) image with the metasurface and a map of enhancement ratio compared to without the metamaterial. Figures reproduced from [77]

The metasurface was experimentally realized by placing the copper strips on a very thin plastic substrate, which was placed in a water pad sealed in a flexible plastic container. The flexibility of this structure allows close contact with the calf. The experiment was performed using a transmit/receive double-tuned birdcage coil. ^1^H scanning comprised a gradient echo sequence with a low-tip angle excitation. A CSI (chemical shift imaging) pure phase encoded method was used for ^31^P spectroscopy. Figure 17 shows experimental imaging and spectroscopy results from a human calf muscle. The maximum enhancement ratios of the SNR, for the same excitation tip angle, were calculated from the images as 1.8 for ^31^P and 2.1 for ^1^H.

**Fig. 18:**
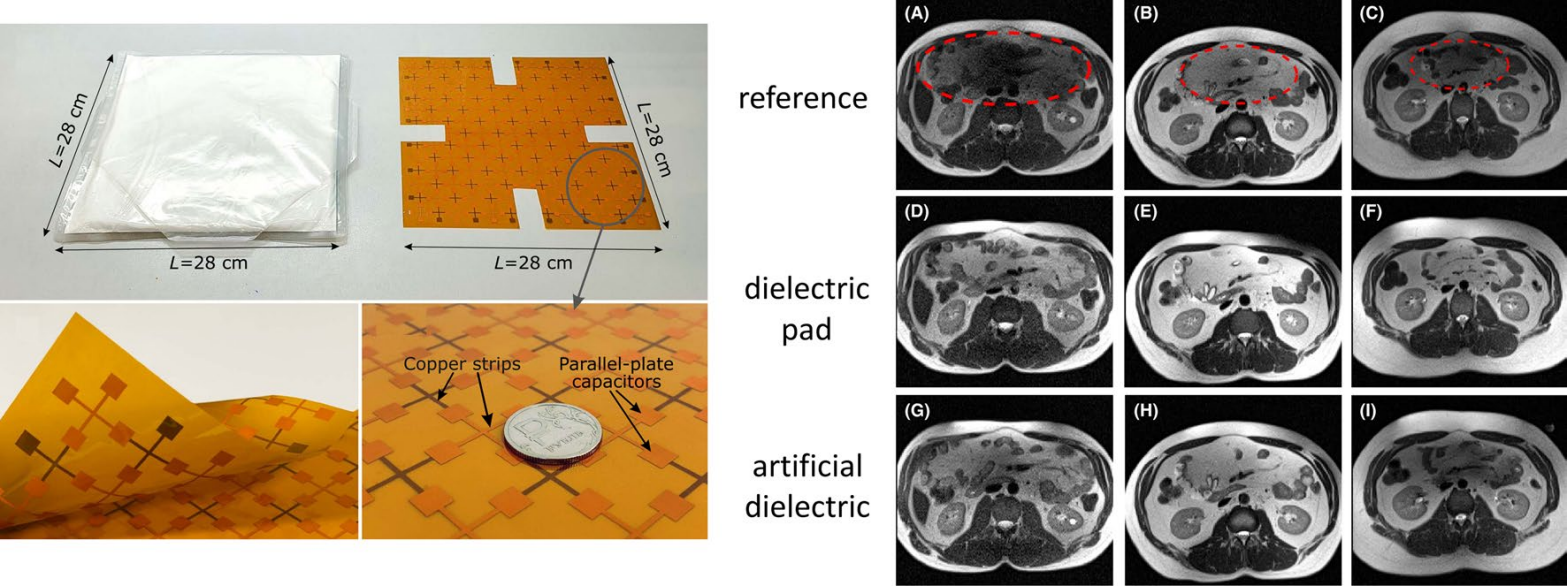
(Left) Photographs of a barium-titanate based dielectric pad (*ε*_r_ ~ 300) and an artificial dielectric/metasurface etched from a flexible double-sided PCB. (Right) 3 T MR images of three healthy volunteers. Figures reproduced with permission from Ref. [82]

These approaches using metasurfaces can produce high effective permittivities, but still rely on having integrated HPMs. Theoretically one can also produce very high “effective permittivities” using purely conductive elements, so-called “artificial dielectrics” [78–80]. Alena Shchelokova’s group at ITMO performed simulations of such materials for 7 T, with the first structure comprising a layer of several printed circuit boards (PCBs) of square copper patches on a thin dielectric substrate designed for 7 T [81]. This artificial dielectric mimicked the action of an equivalently-sized dielectric pad with *ε*_r_ = 120, but was not ideal in terms of the orientation of the secondary B-field created, and the fact that several PCB layers were needed which gave rise to a very rigid structure. They recently showed an improved design approach for abdominal imaging at 3 T [82] using an artificial dielectric produced on a thin single double-sided low-loss PCB with capacitors formed by the overlap of conductive patches on either side of the flexible PCB connected by thin copper strips. Results, shown in Fig. 18, indicated similar improvement in image quality as for a much heavier dielectric pad. Images using the artificial dielectric for the central column in Fig. 18 are slightly better (less hyperintensity at the top) but are slightly worse in the right hand column (greater signal dropout).

## New materials in designing transmit and receive resonators

**Fig. 19:**
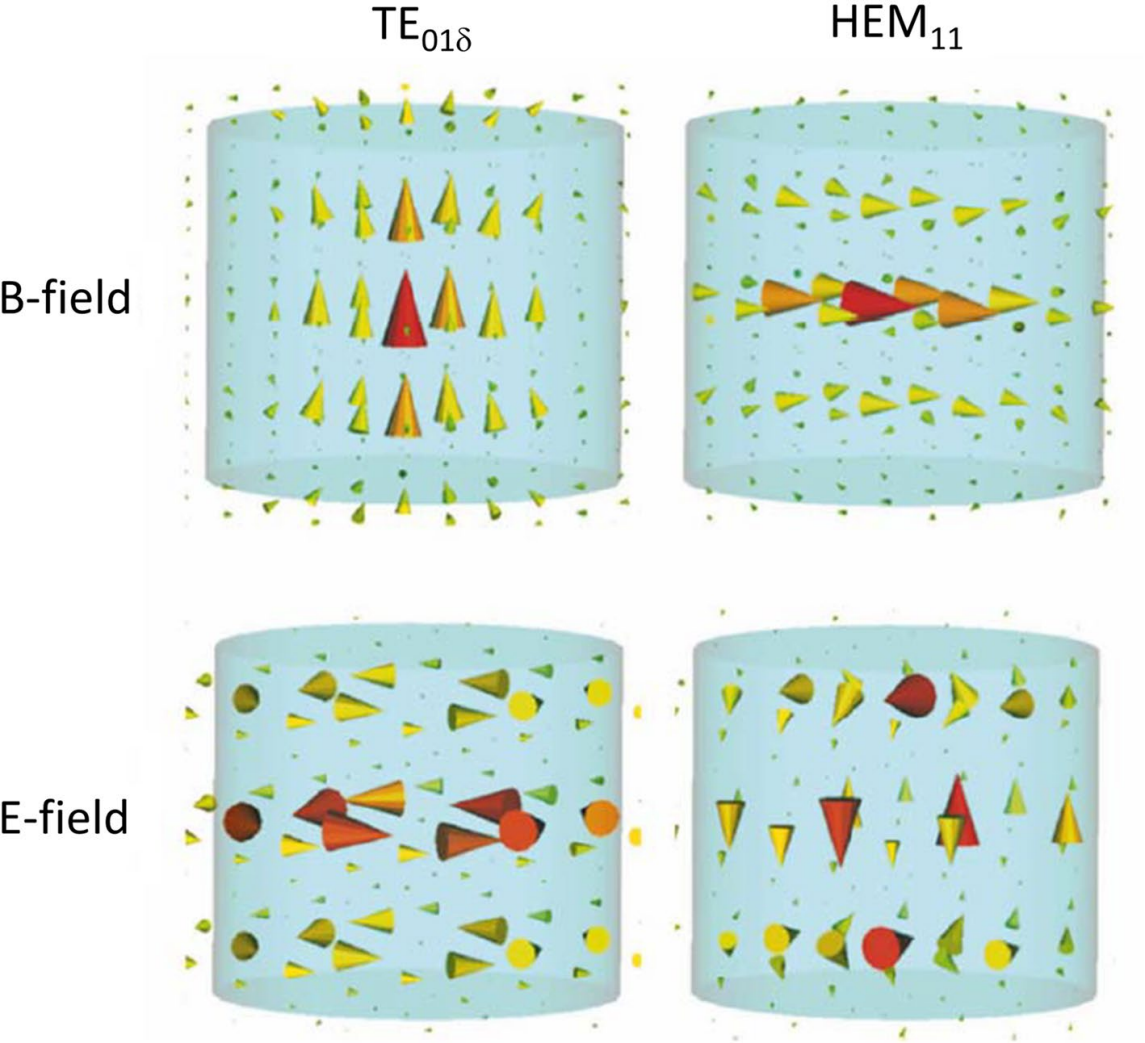
Schematics of the distribution of the magnetic (H) and electric (E) fields in a cylindrical dielectric resonator for the lowest frequency TE and HEM modes

In parallel with the work on different forms of HPM “inserts”, we also realized that these new ceramics which were being produced could also be used as resonators per se, and might have advantages over conventional lumped-element coils. Such resonators, normally referred to as a dielectric resonators (DRs), have a long history in electron paramagnetic resonance (EPR) [83], and we had also previously worked with them in a few high field MR microimaging applications [84]. A DR can support a variety of eigenmodes, the frequencies of which depends on the resonator geometry and the HPM used. Such modes are divided into three categories: transverse electric (TE), transverse magnetic (TM), and hybrid electric and magnetic (HEM). Generally, lower frequency TE and HEM modes are the most useful for MRI experiments due to their EM field distributions. For example, the linear TE_01δ_ mode of an annular resonator produces a magnetic field distribution similar to a solenoid, but with zero electric field in the centre. For the same structure there are two frequency-degenerate orthogonal HEM_11_ modes which produce a magnetic field distribution similar to that of a birdcage coil. The TE_01δ_ and HEM_11_ modes are shown schematically in Fig. 19.

**Fig. 20:**
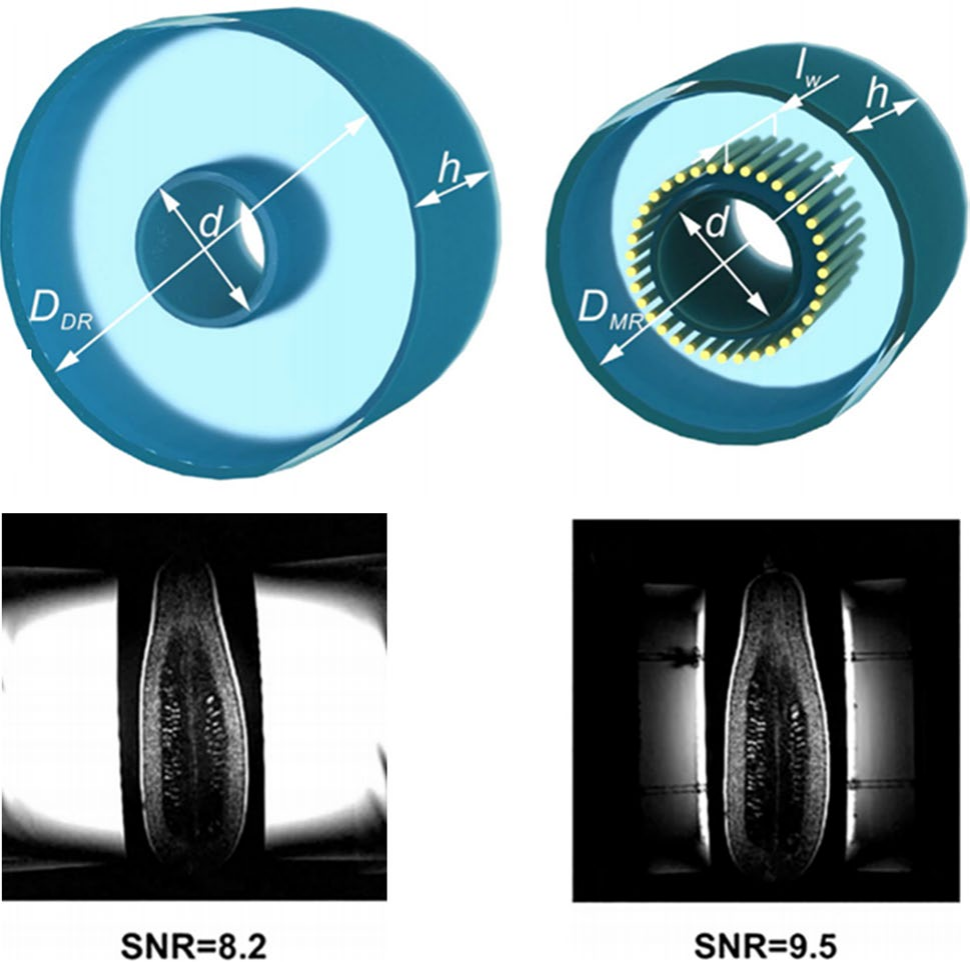
Schematic view of the geometries of (left) an annular dielectric resonator and (right) an artificial-dielectric resonator. The inner diameter *d* = 110 mm and height *h* = 232 mm are equal for both structures, while the outer diameter of the dielectric resonator is 352 mm but the artificial-dielectric resonator’s one is reduced to 220 mm. The length of the wires *l*_w_ is 182 mm. Images acquired from a courgette using the 3 T body transmit coil inductively coupled to the smaller resonators. Figure reproduced from [89]

Using the HEM_11_ field distributions in Fig. 20, Aussenhofer in our group designed a water-based annular dielectric resonator which produces a circularly-polarized magnetic field and was used to image the knee at 7 T [85]. Schmidt et al. adapted this approach to produce a resonator which could be split for increased patient accessibility [86], as well as using HPMs in place of water to produce a compact resonator for ^31^P at 7 T [87]. If a physically smaller resonator is desired then either the actual or the effective permittivity must be increased. Aussenhofer produced a ceramic-based resonator with much higher permittivity than water to image the finger [88].

A natural progression was to see if we could take this concept to the more clinically-relevant 3 T field strength, but we knew that the resonator would become extremely large. Fortunately the concept of artificial dielectrics could be invoked to help the situation. Mikhailovskaya et al. showed a reduction in outer diameter of 37% [89] for a water-based resonator designed for 3 T. When used in an inductively coupled wireless mode, the sensitivity of the artificial-dielectric resonator was measured to be essentially identical to that of a standard dielectric resonator operating in its degenerate circularly-polarized HEM_11_ modes, as shown in Fig. 20.

Circularly polarized EM fields intrinsic to the HEM_11_ mode are very appropriate to many imaging applications, but there are also physical setups in which the field from the TE_01δ_ mode is more appropriate. Shchelokova et al. [90] demonstrated a wireless, inductively coupled DR for breast imaging at 3 T using a material (*ε*_r_ ~ 1000) based on a mixture of BaSrTiO_3_ doped with Mg. This approach was further used for bilateral breast imaging [91] where the system of two coupled dielectric resonators operated in a hybrid TE_01δ_ mode was used.

**Fig. 21:**
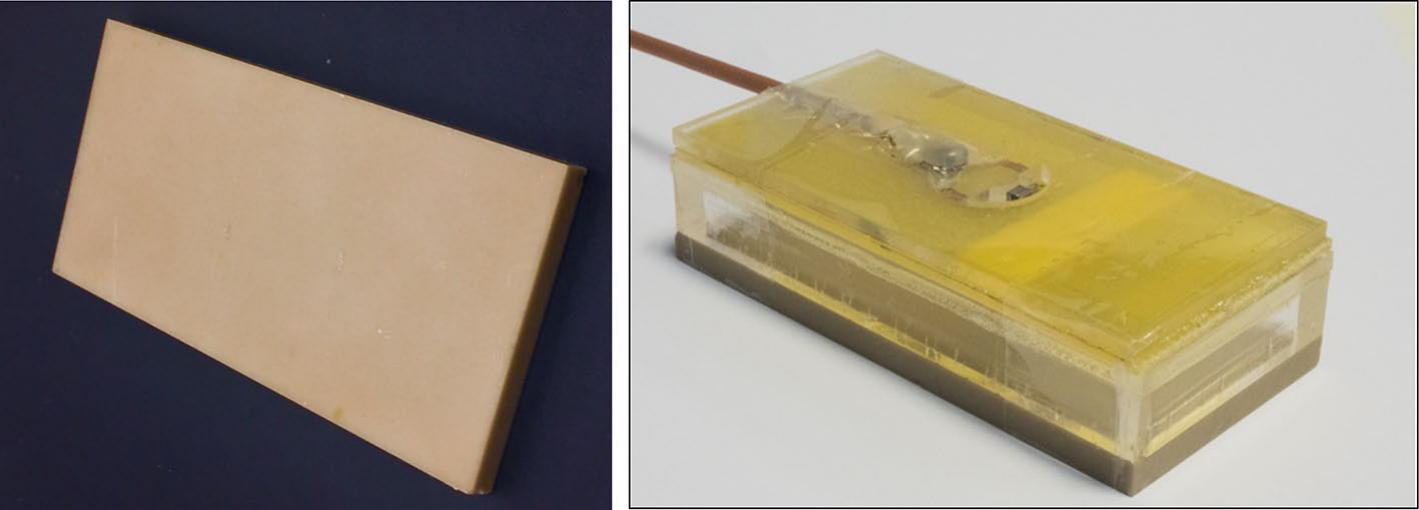
(Left) Dielectric resonator made from PZT with a relative permittivity of ~ 1070. The dimensions of the block are 90 × 44 × 5 mm^3^ so that the frequency of the TE_01δ_ mode is at 298 MHz (7 T). (Right) The DR is coupled inductively to a small loop for transformer impedance matching. Figure adapted from [92]

Transmit and receive arrays can also be constructed using DRs operating in the TE_01δ_ mode, with HPMs, enabling the use of thin elements. For example, a thin rectangular DR produces a magnetic field very similar to that of an equivalently-sized surface coil without the need for extensive capacitive segmentation at high fields. O’Reilly et al. [92] designed DRs using small and light rectangular elements with *ε*_r_ ~ 1070, shown in Fig. 21.

**Fig. 22:**
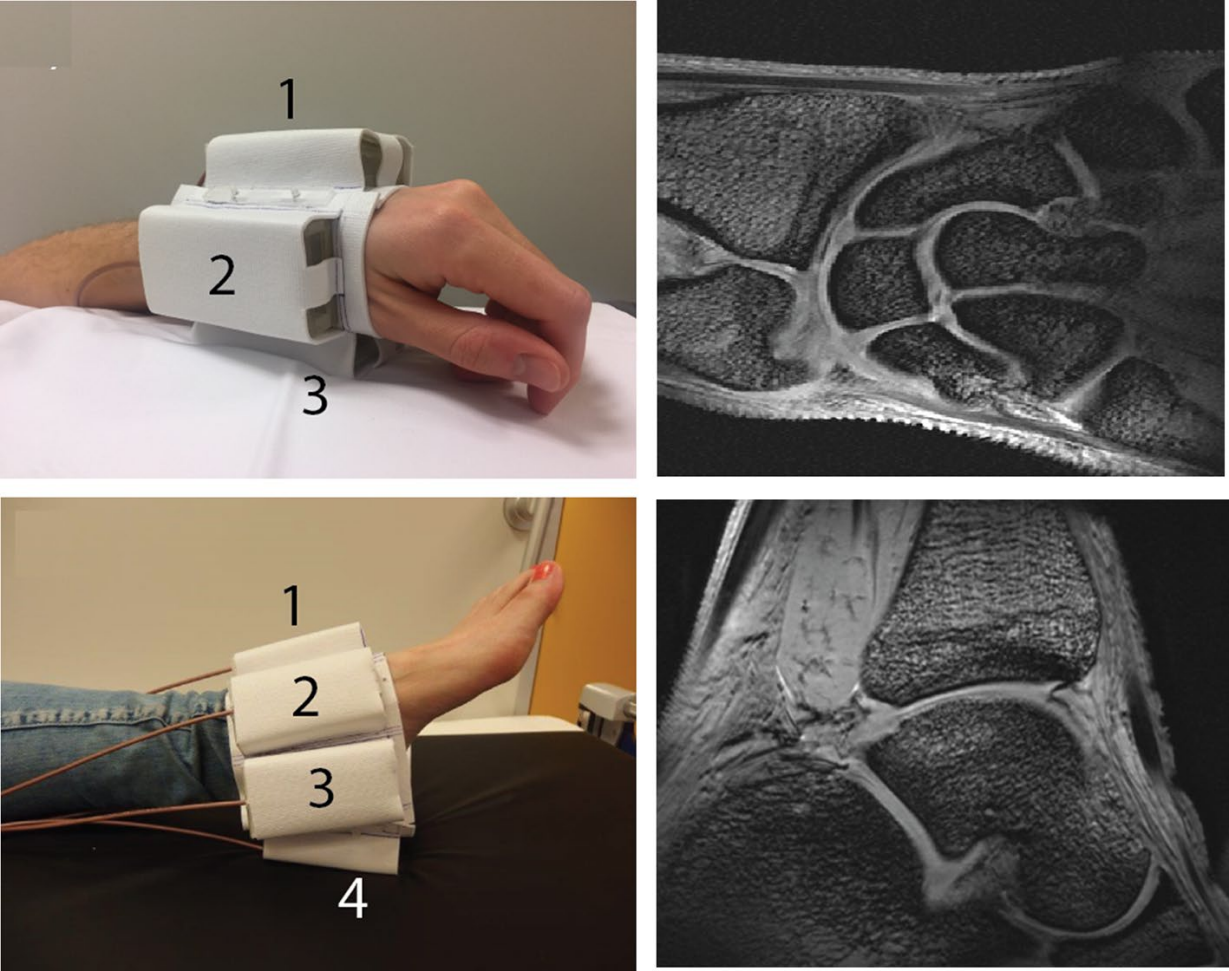
(Top row) An array of four rectangular DRs for imaging the wrist at 7 T: T_1_-weighted 3D gradient echo image acquired using the array in transceiver mode. (Bottom row) An array of eight rectangular DRs: T_1_-weighted 3D gradient echo image obtained using the array in transceive mode. Figure adapted from [92]

In addition to their physical simplicity, we had the idea that DRs should not couple very strongly to each other, and so could be placed close together to allow the construction of flexible transceiver arrays with an arbitrary number of antennas that can be placed conformally on the patient [93]. Figure 22 shows in vivo T_1_-weighted 3D gradient echo images of the wrist and ankle with four and seven DR elements, respectively. No retuning of the DR array elements was required for the different imaging configurations.

**Fig. 23:**
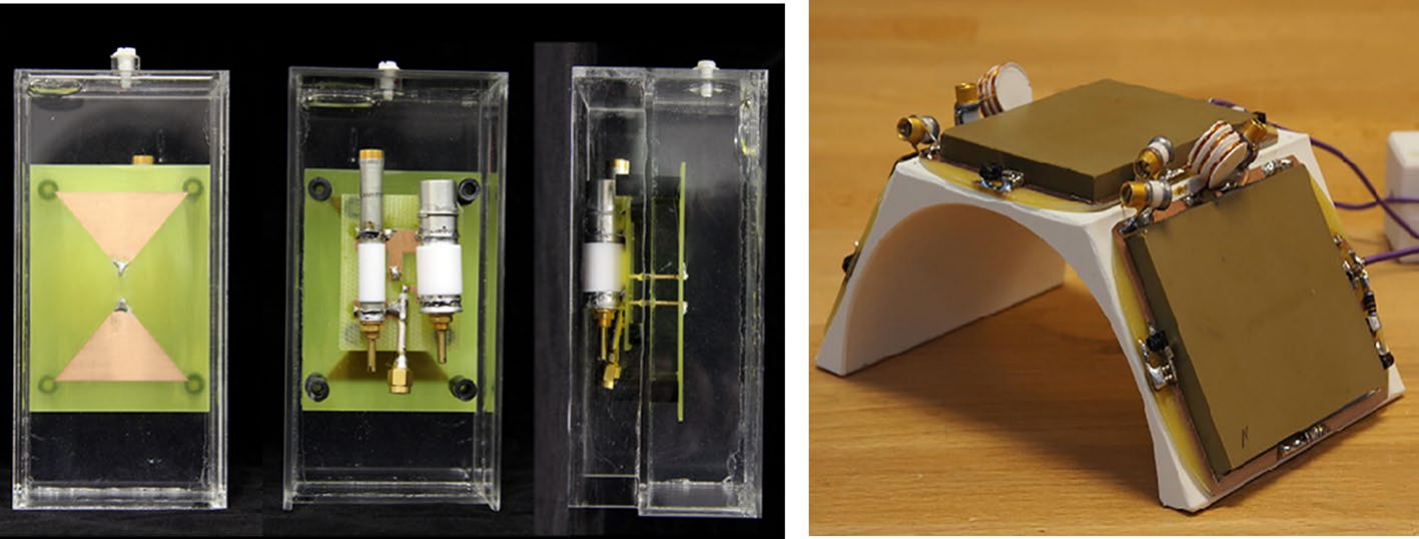
Examples of integration of high permittivity materials into the RF coil itself. (Left) Water (or deuterated water) is used to reduce the electrical length of a bow-tie dipole antenna used for cardiac imaging at 7 T [101], (right) HPMs integrated into a receive coil array used for imaging the larynx at 3 T [99]

Several other groups have also considered various aspects of DR operation at high fields. Wenz et al. [94] have investigated the different modes of a DR that can be excited using a dipole feed, and why certain configurations preserve (and others do not) the transmit field distribution and efficiency if there is a gap between resonator and subject [94]. The group in Utrecht have used HPMs to improve the B_1_^+^ penetration and reduce the SAR of various forms of dipole antenna [95, 96]. The shortened RF wavelength in HPMs can also be used to reduce the physical size of different types of dipole, one example being a short bow-tie antenna used for body imaging at 7 T in which the antenna is submerged in water or deuterated water [97], as shown in Fig. 23a. Dielectric waveguides, which use leaky modes for imaging, have also been constructed from HPMs [98]. HPMs have been integrated into receive-only surface coils at 3 T [99], Fig. 23b, and show improved SNR performance compared to equivalently-sized conventional surface coils. Most recently, a new approach has used a non-uniform dielectric substrate (NODES) design for cardiac imaging at 7 T, in which a non-uniform spatial and permittivity arrangement is placed underneath a shortened dipole and optimized either for transmit or receive performance [100].

As outlined in the introduction, having seen how the design of new types of high permittivity material could enable this approach to be used at clinical field strengths, it became clear that this approach could also be used for very high field MR microscopy. This is particularly attractive since coil losses often dominate when using very small RF coils at high frequencies, and the very thin wire used for millimetre-sized coils has a much higher equivalent series resistance than a very low loss ceramic. As shown in Fig. 19 the TE_01_ mode has an axial magnetic field which has a maximum in the center along the axis of symmetry [102], which also coincides with a minimum electric field. If a hole is made in the centre of the material to produce a ring resonator [84, 103] the sample can be placed in the hole, and the noise contribution from a conductive sample is minimized with the noise being driven by the loss factor of the dielectric material. Early work in MRM with DRs using materials such as barium strontium titanate (Ba_0.04_Sr_0.96_TiO_3_, *ε*_r_ 323) [104] at 600 MHz and calcium titanate (*ε*_r_ 150) [84] at 900 MHz showed a higher sensitivity than saddle coils of similar dimensions, but no detailed simulations or quantitative comparisons were made with, for example, high sensitivity solenoids which are typically used in such experiments.

**Fig. 24:**
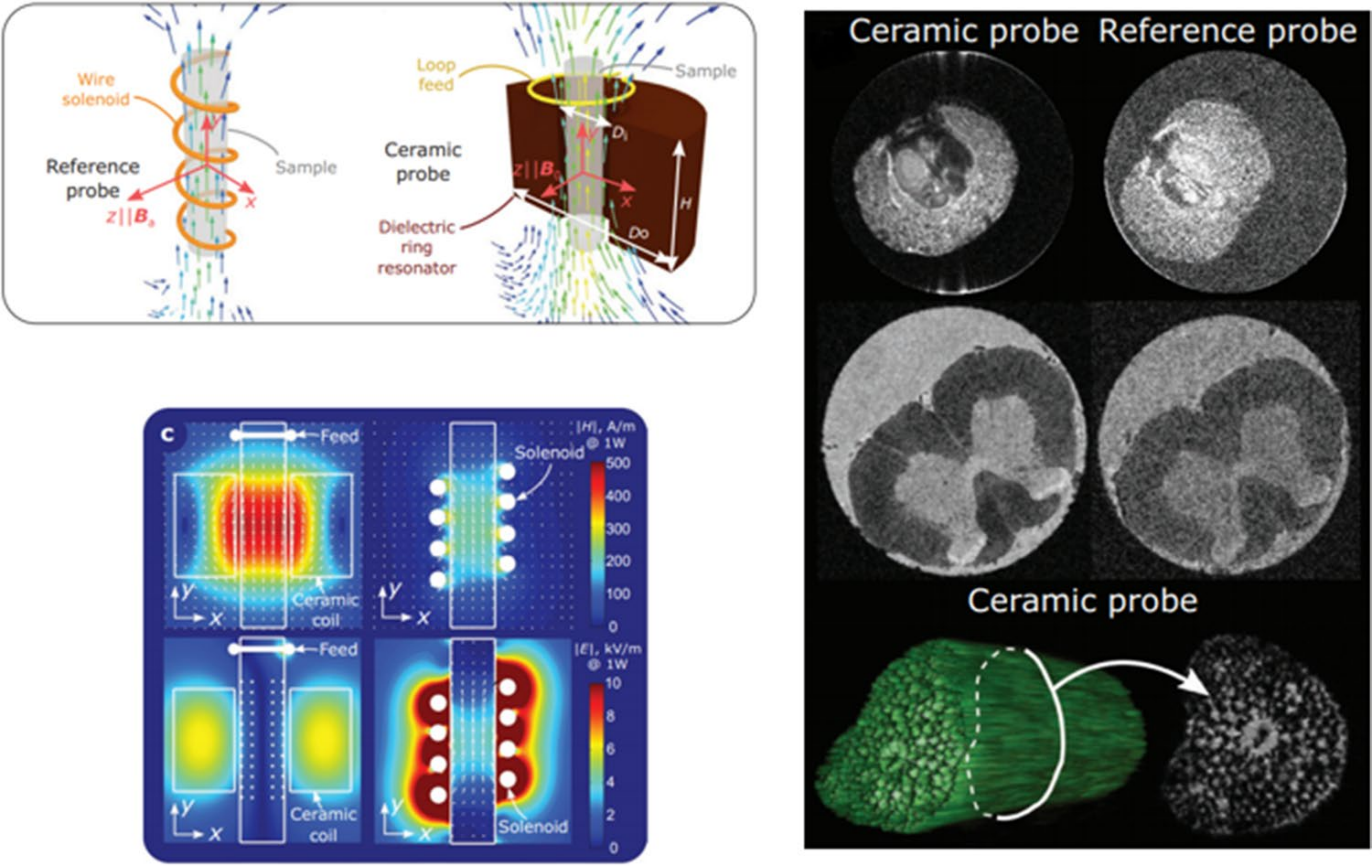
(Top left) Schematics of the magnetic fields created by a solenoid probe and a ceramic probe based on the fundamental mode in an annular-ring resonator. (Bottom left) calculated H-fields (first line) and E-fields (second line) of compared probes at accepted power of 1 W, in the phantom filled with commercial rat-brain solution (permittivity *ε*_r_ = 50 and conductivity *σ* = 1 Sm^−1^). (right) comparison of microscopy images obtained using the ceramic and reference probes (color intensity is normalized by the maximum of the image signal in each case): Ilex aquifolium fruit—first line; chemically fixed rat spinal cord middle line; 3D rendering and image slice of a plant petiole—third line. Figures were reproduced from Ref. [103]

Working with the groups of Luisa Ciobanu in Paris, Stefan Enoch and Redha Abdeddaim in Marseilles and Stas Glybowski in St.Petersburg, DRs were constructed for operation at 17.2 Tesla using new low loss ferroelectric composite ceramics [103], with simulations demonstrating an enhanced SNR by a factor of more than 3 for a biological tissue-mimicking sample material compared with the optimal solenoid. An experimental demonstration of this work showed an enhancement factor of 2.2, slightly lower than the theoretical value. Simulations and experimental results are shown in Fig. 24. The approach can be extended using two coupled DRs, which were shown to be able to simultaneously image two samples with high sensitivity [105].

## Discussion and outlook

Traditional RF coil designs have been based on symmetric arrangements of conductors, with lumped capacitive and inductive elements used for frequency tuning and impedance matching. Geometries such as the birdcage, loop and solenoid are ubiquitous in human, animal and microimaging applications. These coils perform extremely well in terms of homogeneity (birdcage/solenoid) and sensitivity (all coils) when the body/sample does not introduce significant loading effects and alter the transmitted EM field distribution. However, when the imaged dimensions are of the same order of magnitude as the wavelength inside the tissue, then significant distortions in the B_1_ transmit and receive fields occur. In this case use of new materials discussed in this review can make a significant improvement in image quality.

As mentioned in the introduction, dielectric pads were originally designed as a simple temporary solution to the issues of B_1_^+^ inhomogeneity (over 40 sites worldwide currently use these materials) while the subtleties of parallel transmit were being investigated, and the necessary hardware and software were being implemented on commercial systems. Certainly there has been significant progress on that front recently, and the ultimate success would in some ways be the phasing out of the use of dielectric pads in high field MRI. The design of universal pulses, for example, holds the promise of achieving the reproducible and well-characterized nature that is necessary for clinical studies. This will also hopefully also persuade the manufacturers not to build in enormous safety-levels which currently mean that parallel transmit is simply not a practical approach for real clinical applications.

In addition to the effects on the transmit side, HPMs have also been shown to change the reception profile and sensitivity. Much work performed by the New York group [35, 37] has shown that the SNR can be enhanced locally using such HPMs, and that the performance of a multi-channel array becomes similar to one with a larger number of elements if the positions of the HPMs are chosen appropriately, therefore more closely approaching the ultimate SNR. It may well be that the ultimate setup for ultra-high field MRI incorporates parallel transmit arrays, universal pulses, and receiver arrays consisting of different combinations of loops, dipoles and appropriately placed HPMs.

In the field of very high field (> 20 T) MR microscopy, the role of ceramic resonators also looks very promising. Here the main challenge, due to the very high Q value, is the sensitivity of the resonance frequency to temperature. Temperature-compensated ceramics already are used in most capacitors (designation NP0) used in conventional MRI coils, and so one of the next practical steps would be to incorporated such ceramics, perhaps coupled to the inherent temperature control units associated with most high resolution NMR systems, to provide a platform suitable for long timescale scanning protocols.
